# 2D LDH-MoS_2_ clay nanosheets: synthesis, catalase-mimic capacity, and imaging-guided tumor photo-therapy

**DOI:** 10.1186/s12951-020-00763-7

**Published:** 2021-02-03

**Authors:** Jiayan Zhao, Hang Wu, Jiulong Zhao, Yichen Yin, Zhilun Zhang, Shige Wang, Kun Lin

**Affiliations:** 1 Department of Gastroenterology, Changhai Hospital, Second Military Medical University, No. 168 Changhai Road, Shanghai, 200433 People’s Republic of China; 2 College of Science, University of Shanghai for Science and Technology, No. 334 Jungong Road, Shanghai, 200093 People’s Republic of China; 3 Department of General Surgery, Xinhua Hospital, Shanghai Jiaotong University School of Medicine, No. 1665 Kongjiang Road, Shanghai, 200433 People’s Republic of China

**Keywords:** LDH, MoS_2_, Chlorin e6, Catalysis, Tumor therapy

## Abstract

Owing to the hypoxia status of the tumor, the reactive oxygen species (ROS) production during photodynamic therapy (PDT) of the tumor is less efficient. Herein, a facile method which involves the synthesis of Mg–Mn–Al layered double hydroxides (LDH) clay with MoS_2_ doping in the surface and anionic layer space of LDH was presented, to integrate the photo-thermal effect of MoS_2_ and imaging and catalytic functions of Mg–Mn–Al LDH. The designed LDH-MoS_2_ (LMM) clay composite was further surface-coated with bovine serum albumin (BSA) to maintain the colloidal stability of LMM in physiological environment. A photosensitizer, chlorin e6 (Ce6), was absorbed at the surface and anionic layer space of LMM@BSA. In the LMM formulation, the magnetic resonance imaging of Mg–Mn–Al LDH was enhanced thanks to the reduced and acid microenvironment of the tumor. Notably, the ROS production and PDT efficiency of Ce6 were significantly improved, because LMM@BSA could catalyze the decomposing of the overexpressed H_2_O_2_ in tumors to produce oxygen. The biocompatible LMM@BSA that played the synergism with tumor microenvironment is a promising candidate for the effective treatment of cancer.

## Introduction

The methodology of the detection and medical treatment of cancer has seen a rapid growth over the past few decades [1–3]. However, standard clinical therapies of cancer remain with many defects and individual bottlenecks. Surgical resection would cause operative wounds, X-ray exposure may bring serious side effects to healthy tissue because of off-target [4, 5], and chemotherapy usually shows limited efficacy with severe multi-drug resistance [6–8]. Thence, the malignant tumor is still posed as one of the greatest enemies of public health [9, 10], and novel tumor therapeutic approaches to maximizing the treatment efficiency and minimizing the trauma to normal tissue were actively designed. Photon induced tumor therapy, including photothermal therapy (PTT) and photodynamic therapy (PDT), has gained increasing interests in recent years. Tumor PTT relies on the foundation of a photo-thermal transforming agent (PTA) which is capable of transferring the near-infrared (NIR) laser into heat [11–14]. Promisingly, MoS_2_ has been extensively explored as a PTA owing to its facile fabrication, admirable bio-compatibility, and high photo-thermal conversion efficiency [15, 16]. As another aspect of photon-induced tumor therapy, PDT primarily involves the killing of cancer cells using the cytoxic reactive oxygen species (ROS), like singlet oxygen (^1^O_2_). The ROS could be produced when the photosensitizer (PS) and intra-tissue oxygen are irradiated by external light [17]. The applications of different kinds of nano-platforms such as gold nanomaterials [18–21], graphene [22–24], and conjugated polymer-composites [25] to load PS (e.g., chlorin e6, Ce6) for the synergistic tumor PDT and PTT has been extensively studied. However, owing to the hypoxia status of tumors, the ROS production is limited, leading to an unsatisfactory tumor PDT efficiency [26, 27].

As a kind of frequently studied bio-degradable contrast agent, manganese-based nanomaterials could produce numerous Mn(II) paramagnetic centers to enhance the T_1_-weighted MR imaging performance of tumors [28, 29]. More importantly, manganese-based nanomaterials could catalyze the decomposing of the over-expressed hydrogen peroxide (H_2_O_2_) to generate oxygen in tumor and alleviate the tumor hypoxia conditions [30]. Many kinds of clay materials are characterized with layer structure and the interlayer space of the clay has been extensively studied for effective drug encapsulation [31]. Layered double hydroxides (LDHs), accompanying with particular physical properties such as high surface area, acidity, and structural stability, is a class of two dimensional (2D) anionic nano-clay with positive-charged layers [32–34]. The internal galleries of LDHs could exchange with other ions in the external environment [35]. Moreover, their physicochemical properties could be adjusted by modulating the ratio of metallic cations and the sort of interlayer anions [36–38]. Recently, the fabrication of LDH-based clay nanocomposites has gained ever-increasing interests. For example, copper (Cu(II)) or cobalt (Co(II)) sulfamerazine–salicylaldehyde complexes were used to intercalate the Mg-Al-LDH that was synthesized by a co-precipitation route [39]. In this research, Mg-Al-LDH composite, which exhibited promising antimicrobial activity against both gram-positive (*Staphylococcus aureus*) and gram-negative (*Escherichia coli*) bacteria, was prepared via a two-step delaminating/restacking method. These motivate us to take the challenge of fabricating an LDH-based composite nano-platform to incorporate the photo-thermal effect of MoS_2_ for the imaging-guided combined tumor therapy.

Herein, a facile hydrothermal synthesis of LDH-MoS_2_ (LMM) clay nanosheets was proposed. The LMM nanosheets were then surface coated with bovine serum albumin (BSA) to gain the colloidal-stability and biocompatibility in vitro and in vivo. Protein/peptide has been frequently used as the template for the biomimetic mineralization, and has been demonstrated to be an efficient and promising strategy for synthesis of nanoparticles for bioapplications [40]. In this LMM@BSA formulation, the photo-thermal transforming agent (i.e., MoS_2_) was distributed on the surface and anionic layer space of LDH anionic layer-space the as-designed LDH. MoS_2_ provides the possibility to suppress the tumor cell malignant proliferation via NIR laser-induced hyperthermia. At the same time, Mn element renders the LMM@BSA clay nanosheets the tumor reducibility and acidity responsive MR imaging and catalase-mimic capacities to catalyze the disproportionation of H_2_O_2_. The generated oxygen could alleviate the tumor hypoxia conditions to enhance the production of ROS during PDT. The LMM@BSA clay nanosheets were used to efficiently load the photosensitizer Ce6, which could play the synergism with the catalase-mimic capacity of LMM@BSA to enhance the tumor PDT efficiency. To the best of our knowledge, the synthesis of LDH-based nanocomposites with imaging capacity for combined tumor photo-therapy has not been reported yet.

## Experimental section

### Synthesis of LMM@BSA clay nanosheets

All the distilled water applied in this study with a resistivity higher than 18.2 MΩ was produced using a Milli-Q Plus 185 water purification equipment (Millipore, Bedford, MA). The layered LMM nanosheets were synthesized via a hydrothermal method. Firstly, 0.164 g Mg(NO_3_)_2_·6H_2_O (Adamas-beta, Shanghai, China), 0.04 g Mn(NO_3_)_2_·4H_2_O (Adamas-beta, Shanghai, China), and 0.06 g Al(NO_3_)_3_·9H_2_O (General Reagent, Shanghai, China) were dissolved together in 10 mL distilled water. The formed aqueous solution was quickly mixed with 20 mL NaOH (Aladdin, Shanghai, China, prepared into 0.15 mol/L solution). After one hour of magnetic stirring at room temperature, the solution was centrifuged (8000 rounds/min, 5 min) to collect the sediment. The sediment was then dissolved into 25 mL distilled water and then mixed with 10 mL (NH_4_)_2_MoS_4_ (J&K Chemical, Shanghai, China) aqueous solution (5 mg/mL) and magnetically stirred for 1.5 h (600 rounds/min). After sealed into a 100 mL stainless steel autoclave that lining with polyphenylene, the mixture was heated in an oven at 180 °C for 12 h. Then, the product was thoroughly water-washed for three times and centrifuged (8000 rounds/min, 5 min) to get LMM clay nanosheets. The Mg-Al-LDH nanosheets preparation was similar to the synthesis of LMM nanosheets but without the addition of Mn(NO_3_)_2_·4H_2_O. The sediment was dissolved into 35 mL distilled water. The formed LMM clay nanosheets and Mg-Al-LDH nanosheets were lyophilized for future use. To prepare LMM@BSA clay nanosheets, 10 mg freeze-dried powder of LMM was dispersed in 10 mL distilled water with 250 mg BSA (Aladdin, Shanghai, China). The product then underwent an ultrasonic shattering (500 W, 120 min), centrifugation (13,000 rounds/min, 10 min), and twice water-washed. The product was dissolved into 10 mL distilled water for further application.

### Material characterization

The surface morphology of LMM nanosheets was recorded using a scanning transmission electron microscopy (SEM, FEI Magellan 400). The microstructure of LMM@BSA clay nanosheets was observed by a transmission electron microscopy (TEM, FEI Tecnai G2 F20) and the thickness of LMM@BSA nanosheets was determined using an atomic force microscope (AFM, Bruker Dimension ICON). Before the SEM and TEM observation, LMM nanosheets was dissolve in water with a concentration of 100 μg/mL. The distribution of Al, Mg, Mo, Mn, O and S in the LMM nanosheets was mapped with the Energy-dispersive X­ray spectroscopy (as the accessory of TEM). The chemical nature of LMM material was characterized by X-ray photoelectron spectroscopy (XPS, Thermal Scientific ESCAlab250). The test results were calibrated by the C1s peak (284.8 eV). X-ray diffraction (XRD, Rigaku D/max-2200 PC) system was used to assess the crystalline structures of LDH and LMM nanosheets [operation parameters: Cu Kα radiation, the wavelength at 1.54 Å, scanning from 5° to 70° (2θ)]. The scanning voltage and current was set as 40 kV and 40 mA, respectively. The chemical information of BSA and freeze-dried powder of LMM and LMM@BSA was determined by a Fourier Transform Infrared (FTIR) spectroscopy under the transmission mode in the wavelength range of 4000 to 500 cm^−1^ (Nicolet 7000-C spectrometer). The dynamic light scattering (DLS) diameters of LMM@BSA clay nanosheets in various solutions were also measured (Malvern Nano ZS90 Zetasizer Nano series). The mass ratio of surface-modified BSA was determined using thermogravimetric (TG209F1 system, NETZSCH Instruments Co., Ltd., Germany). Samples were heated from 50 to 900 °C with a heating rate of 20 °C/min under air atmosphere. The UV–vis–NIR spectrometer (Lambda 25, Perkin Elmer, USA) was used to record the light absorption of nanosheets.

### In vitro photo-thermal performance

The in vitro photo-thermal performance of the LMM@BSA clay nanosheets was studied by continuously irradiating the nanosheets solution with 808 nm NIR laser (Shanghai Connet Fiber optics Company). The distance between the sample and the emitting end of NIR laser was 15 cm. To study the influence of materials concentration on the photothermal conversion, a cell culture plate (96-well) of LMM@BSA clay nanosheets solutions at various concentrations (0 (distilled water, control), 50, 100, and 250 μg/mL) was irradiated with NIR laser (1.0 W/cm^2^) for a duration of 5 min. To study the influence of power density on the photothermal conversion, LMM@BSA clay nanosheets (500 μg/mL) were irradiated with NIR laser (0.2 W/cm^2^, 0.5/cm^2^, 0.8/cm^2^ and 1.0 W/cm^2^) for a duration of 5 min. The temperature changes (*△T*) and the related thermal images were recorded using a FLIR™ E60 infrared camera. To prove the photo-thermal stability of the LMM@BSA clay nanosheets, 100 μL solution was irradiated with NIR laser (808 nm), and its *△T*s in 10 cycles (laser on/off in turn) were plotted. The photo-thermal conversion efficiency (η) of the LMM@BSA clay nanosheets was ascertained with a modified Korgel’s research method [41, 42], whose value could be calculated as follows:

1 $$\upeta = \frac{{hS\left( {T_{max} - T_{Surr} } \right) - Q_{in,Surr} }}{{I\left( {1 - 10^{{ - A_{\lambda } }} } \right)}}*100\% .$$

In this formula, *S* denotes the irradiated surface area of nanosheets. *hS* could be determined by measuring the temperature dropping-speed since the beginning of the laser-off. *T*_*max*_ refers to the highest temperature of the nanosheets solution. *T*_*surr*_ is the ambient temperature. *Q*_*in,surr*_ implies the heat transferred to the surrounding. *I* and *A (λ)* respectively represent the laser power (in Watt) and the absorbance of the LMM@BSA clay nanosheets at 808 nm.

### In vitro cytocompatibility

At the incubation conditions (37 °C and 5% CO_2_), L929 cells (bought from Institute of Biochemistry and Cell Biology, the Chinese Academy of Science, Shanghai, China) were cultivated in DMEM which contained 100 μg/mL streptomycin, 100 U/mL penicillin and 10% fetal bovine serum. To assess the in vitro biocompatibility, the L929 cells were seeded into a cell culture plate (96-well, 8 × 10^3^ cells/well). After cultured for 12 h, the old medium was replaced with LMM@BSA clay nanosheets solution (500, 250, 100, 50, and 0 μg/mL (control) in DMEM). After a 24-h incubation, the DMEM and materials were discarded. Cells were washed with PBS for 3 times and the metabolic activity and morphology of the L929 cells were evaluated by a cell counting kit-8 (CCK-8, Dojindo, Japan) and Live/Dead staining (LIVE/DEAD™ Cell Vitality Assay Kit, ThermoFisher Technologies, USA) according to the instructions. The live cells stain in green and the dead cells stain red. The stained cells were photographed using a Leica DM IL LED (Germany) inverted phase-contrast microscope.

Mice red blood cells (mRBCs) were centrifuged from the serum and washed with saline for 3 times. Thereafter, mRBCs were stored in PBS at 4 °C. Upon experiment, 0.2 mL mRBC suspension was evenly dispersed in three 1.5-mL Eppendorf tubes with 0.8 mL distilled water, saline, or LMM@BSA clay nanosheets (in saline) respectively. The final concentrations of the nanosheets are 50, 100, 200, and 500 μg/m. The mixtures were cultured for 2 h at 37 °C and centrifuged (5000 rounds/min, 3 min). The absorbance of the supernatants at 541 nm was detected using the UV–vis–NIR spectrometer (Lambda 25, Perkin Elmer, USA). The hemolytic percentage (HP) was derived in the Eq. (2):

2 $${\text{HP}} \left( \% \right) = \frac{{\left( {A_{t} - A_{nc} } \right)}}{{\left( {A_{pc} - A_{nc} } \right)}}.$$

In this formula, *A*_*nc*_, *A*_*pc*_, and *A*_*t*_ are absorbance values of PBS, water, and LMM@BSA clay nanosheets treated blood supernatant, respectively.

### Ce6 loading and singlet oxygen detection

The LMM@BSA and Ce6 were magnetically stirred for 24 h at room temperature in dark. The final concentration of LMM@BSA was 100 or 500 μg/mL, and the final Ce6 concentration was 10, 50, or 100 μg/mL. Then, the mixture was centrifuged (12,000 rounds/min, 10 min) to separate the superfluous Ce6. The sediment was washed with distilled water for 3 times, and the loading efficiency of Ce6 could be determined according to the UV–vis–NIR spectra absorbance of these liquids, using the standard curve of Ce6 at 403 nm. The loading efficiency was calculated in accordance with the formula (3):

3 $${\text{Loading}}\;{\text{efficiency}} \left( \% \right) = \frac{{{\text{C}}_{0} - {\text{C}}_{{\text{s}}} }}{{{\text{C}}_{0} }}*100\% .$$

In this formula, C_0_ is the total concentration of Ce6, and C_s_ is the Ce6 concentration in the supernatant.

A JPBJ-608 dissolved oxygen analyzer (Shanghai INESA Scientific Instrument Company) was used to quantize the dissolved oxygen (DO) content in LMM@BSA clay nanosheets solution. To this end, the electrode immersed in LMM@BSA clay nanosheets (500 μg/mL) solution with or without H_2_O_2_ (50 mM). These solutions were hand-shaken at 20 – 40 cm/s. The DO values were record by the analyzer at an interval of 30 s in a total duration of 10 min. The singlet oxygen (^1^O_2_) produced by the Ce6 loaded nanosheets was probed using 1,3­diphenylisobenzofuran (DPBF). In detail, 50 µL DPBF (10 mM in ethanol) was added into the LMM@BSA/Ce6 aqeous solution (2.95 mL, 1 mg/mL) with the absence or presence of H_2_O_2_ (final concentration 50 mM). The absorption spectra of the mixed solution were recored every 5 min during the 660 nm laser irradiation (30 min, 0.1 W/cm^2^) using the UV–vis–NIR spectrometer (Lambda 25, Perkin Elmer, USA).

### In vitro tumor therapy

#### In vitro tumor PTT

Human colorectal carcinoma (HT29) cells (obtained from Institute of Biochemistry and Cell Biology, the Chinese Academy of Science, Shanghai, China) were seeded in a 96-well plate (8 × 10^3^ cells/well) containing 100 μL DMEM per well overnight. Next, the fresh medium with LMM@BSA clay nanosheets [0 (PBS), 50, 100, 250, and 500 μg/mL] was substituted for the pure medium and the cells were cultured for 4 h. Then, the cells were irradiated with 808 nm laser for 5 min. To study the influence of power density on the cell viability, the cells cultured with 500 μg/mL LMM@BSA clay nanosheets were cultured for 4 h and then irradiated with 808 nm NIR laser (0.2, 0.5, 0.8, and 1.0 W/cm^2^) for 5 min. The metabolic activity and morphology of the L929 cells were evaluated by a cell counting kit-8 (CCK-8, Dojindo, Japan) and Live/Dead staining.

#### In vitro tumor PTT and PDT

To study the irradiation time-dependent tumor PDT, cells (5 groups) were cultured with LMM@BSA/Ce6 nanosheets (LMM@BSA: 100 μg/mL; Ce6: 10 μg/mL) and cultured for 4 h. Then, the alternated irradiation of 660 nm laser (0.1 W/cm^2^) was applied to the cells (group I: without irradiation, control; group II: 1 min; group III: 2 min; group IV: 3 min; group V: 5 min). To study the combined tumor PTT and PDT, cells were cultured with LMM@BSA clay nanosheets (group I and II, 100 μg/mL) or LMM@BSA/Ce6 (group III and IV, 100 μg/mL LMM@BSA, 10 μg/mL Ce6). Then, the alternated irradiation of 660 nm laser (0.1 W/cm^2^) and 808 nm laser (1.0 W/cm^2^) was applied to the cells (group I: without irradiation; group II: 808 nm, 5 min; group III: 606 nm, 5 min; group IV: 606 nm, 5 min, and 808 nm, 5 min). The metabolic activity and morphology of the L929 cells were evaluated by a cell counting kit-8 (CCK-8, Dojindo, Japan) and Live/Dead staining.

### In vivo biocompatibility

The in vivo studies were performed in Changhai Hospital, Second Military Medical University. The animal-handling was in accordance with the policies of the National Ministry of Health. KM mice (SPF level, Shanghai Slac Laboratory Animal Center, China) were intravenously (I.V.) injected with 200 μL LMM@BSA clay nanosheets solution (3 mg/mL, in saline). Another group injected with 200 μL saline was set as control. These mice were euthanized on the 1st day, 7th day, and 14th day. The body weight of KM mice was about 20 g. Therefore the injected dosage of LMM@BSA clay nanosheets to mice could be determined as about 30 mg/kg. Major organs (kidney, lung, spleen, liver, and heart) of these mice were weighed and aqua regia was used to thoroughly digest these weighed organs for 3 days to quantify their respective contents of Mn ions with an Agilent ICP-OES (700 Series). The KM mice body weights were also monitored during the experiment. Standard hematoxylin–eosin (H&E) dyeing was introduced to assess the in vivo biosafety of the LMM@BSA clay nanosheets with the help of a Leica DM IL LED inverted phase-contrast microscope.

For the in vivo hemocompatibility assessment, the routine blood test (using Sysmex XS-800i automated hematology analyzer) and serum biochemistry test (using Beckman Coulter Unicel DxC 800 automatic biochemical analyzer) were performed as follows: KM mice I.V. administered with 200 μL saline (set as control) or nanosheets solution (3 mg/mL) were anesthetized by puncturing the heart for blood-drawing on the 1st, 7th, and 14th day post-injection.

### In vitro and in vivo MR imaging

The Mn content in 20 mg LMM@BSA clay nanosheets (digested in aqua regia) was firstly ascertained by an Inductive Coupled Plasma Emission Spectrometer (ICP). LMM@BSA clay nanosheets solutions at a gradient Mn concentration (1.0 mL, Mn concentration: 0.2 mg/mL, 0.5 mg/mL and 1.0 mg/mL) in three solvents, namely distilled water, GSH, and citric acid buffer (pH = 5.0) and incubated for 2 h. Then, T_1_-weighed MR imaging was finished on an MR imaging system (GE Signa 3.0 T, imaging parameters: TR = 600 ms; TE = Min Full; bandwidth = 15.63 kHz; and slice thickness = 3 mm).

The nude mice (Shanghai Slac Laboratory Animal Center, Shanghai, China) were subcutaneously injected with 150 μL serum-free DMEM containing 1 × 10^7^ HT29 cells on their backs for the construction of tumor models. When the tumor grew into ~ 0.5 cm^3^ after approximately 2 weeks of feeding, the tumor-bearing mice were intratumorally (I.T.) or I.V. injected with the LMM@BSA clay nanosheets (1 mg/mL in saline). These mice were anesthetized and placed in a home-made plate immediately after the I.T. materials injection or 12 h after the I.V. materials injection for the in vivo imaging (imaging parameters: TR = 400 ms; TE = 8.9 ms; Fov = 6 × 6 cm; and slice thickness = 2 mm). The relative brightness intensity (RBI) was calculated based on the Eq. (4):

4 $${\text{RBI}} = \frac{{{\text{BI}}_{{\text{x}}} }}{{{\text{BI}}_{{\text{o}}} }}.$$

In this formula, BI_x_ and BI_o_ represent the brightness intensity of the experimental and control groups in the MR region of interest (area = 5 mm^2^), respectively.

### In vivo tumor therapy

Tumor-bearing mice were randomized into five groups (n = 3). These mice were I.V. injected with saline (group I, 200 μL), or 200 μL LMM@BSA/Ce6 (group II – IV, 1 mg/mL in saline). Members in group V were I.T. injected with 20 μL LMM@BSA/Ce6 (in saline). After 12 h, the mice in the group II and III were received the 808 nm (group II, 1.0 W/cm^2^, 5 min) and 660 nm (group III, 0.1 W/cm^2^, 5 min) NIR laser irradiation respectively. The mice in group IV and V were successively irradiated with 660 nm (0.1 W/cm^2^) and 808 nm (1.0 W/cm^2^) laser for 5 min. The ΔTs of tumor and the thermal images during treatment were recorded using a FLIR™ E60 camera. At different time points, relative tumor volume (denoted as *V*/*V*_0_, *V*_0_ and *V* represent the real-time and initial tumor volume, respectively) and the appearance of each tumor-bearing mouse was recorded. The power density of 808 nm laser is 1.0 W/cm^2^ and the power density of 660 nm laser is 0.1 W/cm^2^ in this study.

### Drug loading and release

To load DOX, a stock solution of DOX (10 mg/mL, 20 μL) was added into the LMM@BSA clay nanosheets solution (1 mg/mL) under room temperature and stirred for 24 h in the dark. The product was purified by centrifugation (12,000 rounds/min, 10 min) and rinsed with water for 3 times. The absorbance of the collected supernatant at 480 nm was determined by UV–vis–NIR spectroscopy to calculate the DOX loading quantity according to the concentration-absorbance standard curve of DOX at the same wavelength.

The drug release from the LMM@BSA/DOX nanosheets was studied at different pH values and temperatures. In detail, LMM@BSA/DOX solutions were placed in vials containing 5 mL buffer with different pH values [PBS (pH = 7.4) or citrate buffer solution (pH = 6.0)] and incubated at 54 °C or 37 °C, respectively. At pre-designed time points, 1 mL buffer containing the released DOX was taken out and its absorbance at 480 nm was monitored for calculating the real-time released DOX amount. Finally, 1 mL fresh buffer was supplanted.

### Statistical analysis

The one way ANOVA statistical analysis was chosen to determine the significance of data, where 0.05 was appointed as the threshold [(*) p < 0.05, (**) p < 0.01, (***) p < 0.001]. Unless specified, the sample size is three (n = 3) in this study.

## Results and discussions

### Materials synthesis and characterization

**Scheme 1:**
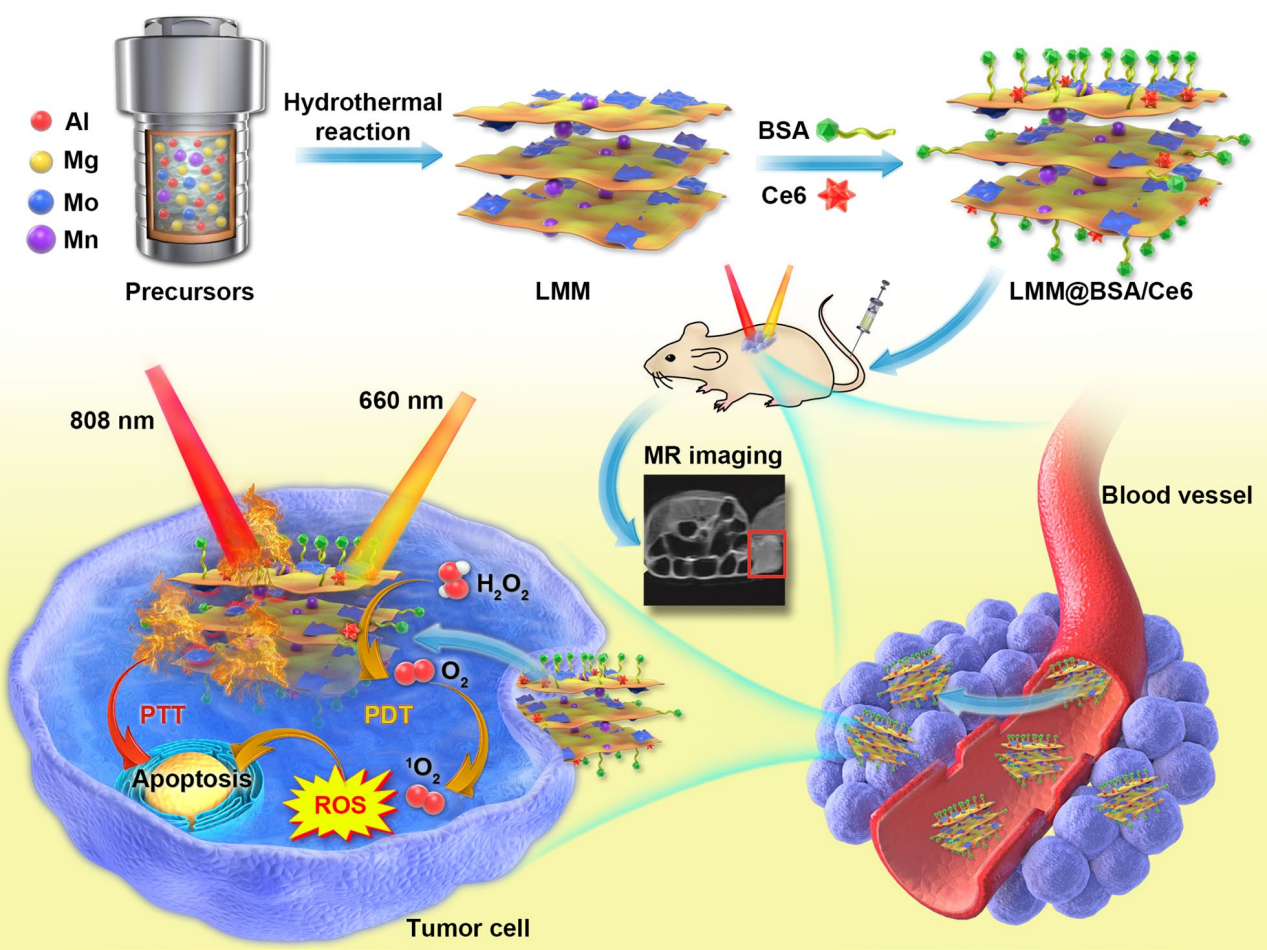
Schematic illustration of the hydrothermal synthesis with BSA coating and Ce6 loading and the synergistic tumor photo-therapy procedure of LMM@BSA/Ce6 nanosheets simultaneously including catalase-mimic and imaging-guided capacity

**Fig. 1:**
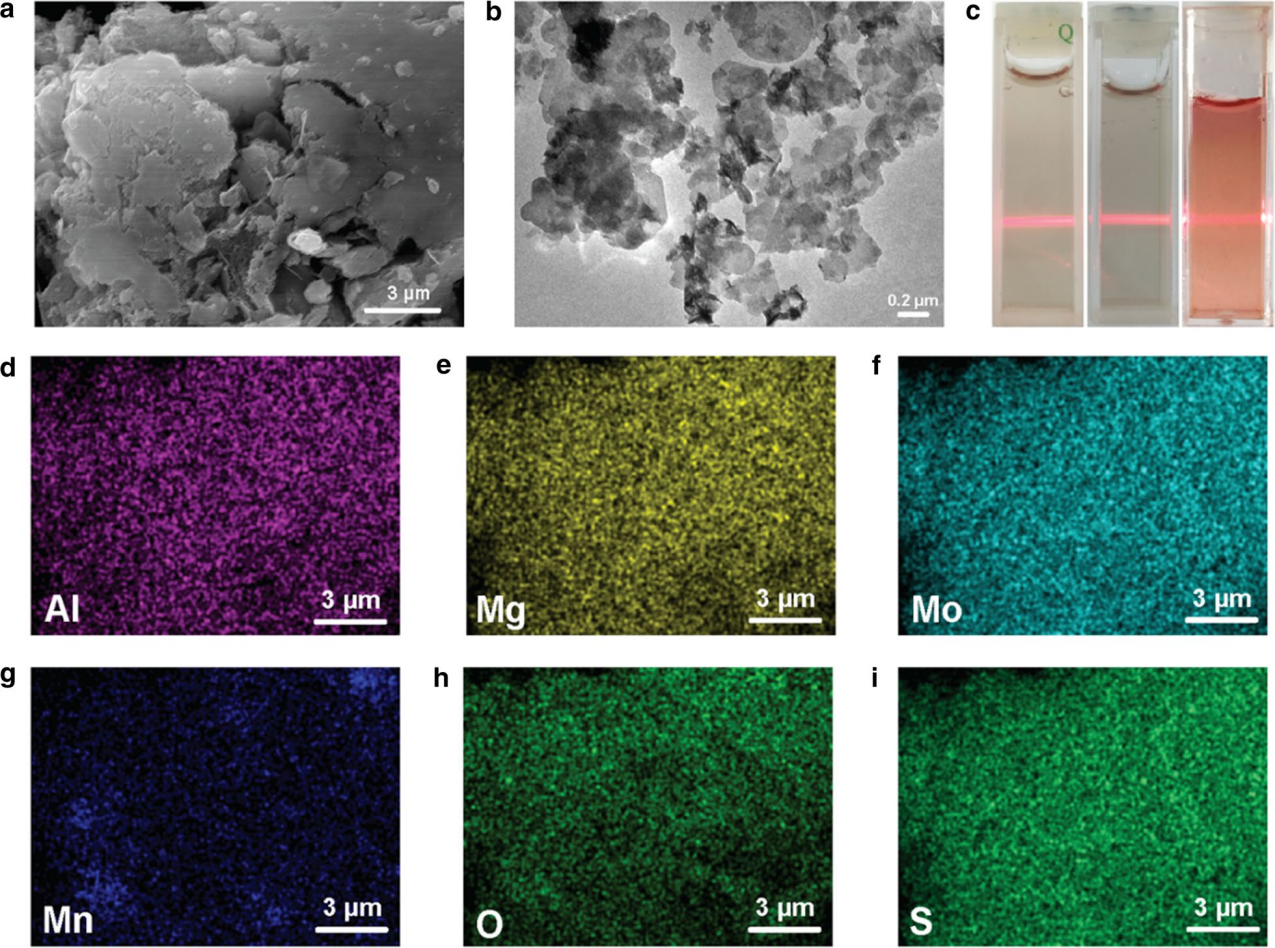
**a** SEM of LMM nanosheets; **b** TEM of LMM@BSA nanosheets; **c** photographic image of typical Tyndall phenomenon of LMM@BSA clay nanosheets in water (left), saline (middle), and DMEM (right); **d**–**i** Al, Mg, Mo, Mn, O and S elemental distribution mappings of LMM nanosheets

Unlike the traditional co-precipitation route which involves the two-step nucleation and sediment ageing [43], the LDH-based clay nanosheets, namely the LMM, was synthesized via hydrothermally treating the mixture solution of NaOH, (NH_4_)_2_MoS_4_, Mn(NO_3_)_2_·4H_2_O, Mg(NO_3_)_2_·6H_2_O and Al(NO_3_)_3_·9H_2_O. During the hydrothermal synthesis, Mn(NO_3_)_2_·4H_2_O, Mg(NO_3_)_2_·6H_2_O and Al(NO_3_)_3_·9H_2_O were transformed into Mg–Mn–Al-LDH, and (NH_4_)_2_MoS_4_ was transformed into MoS_2_. The LMM was sonicated in BSA soluton for BSA coating to gain colloidal stability in physiological conditions. The as-prepared LMM@BSA clay nanosheets were further used to load Ce6, which could generate the cytoxic ROS (i.e., ^1^O_2_) upon the 660 nm laser irradiation, for the MR imaging-guided photo-therapy of the tumor (Scheme 1). It could be easily observed from the SEM that the as-prepared product presents a 2D structure (Fig. 1a, b). After the sonication in BSA solution, LMM@BSA multi-layers with a thickness of ~ 5.5 nm was obtained (Additional file 1: Fig. S1a, b). The element mapping (Fig. 1d–i) further verifies the even coexistence of Al, Mg, Mo, Mn, O, and S in the LMM@BSA clay nanosheets.

**Fig. 2:**
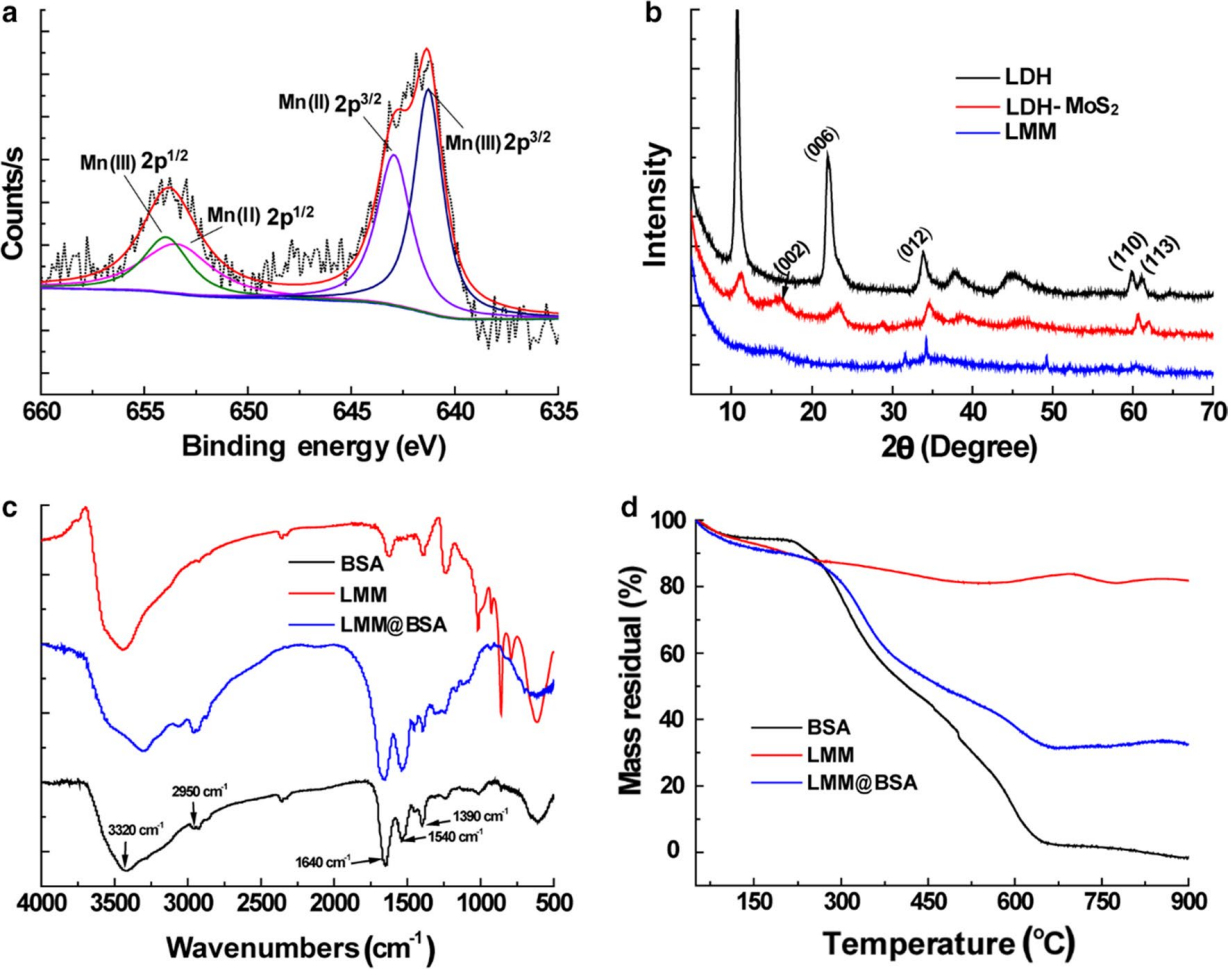
**a** XPS spectrum of Mn 2p; **b** XRD patterns of LDH, LDH-MoS_2,_ and LMM nanosheets; **c** FTIR spectra of LDH, LMM, and LMM@BSA nanosheets; **d** TG curves of LDH, LMM_,_ and LMM@BSA nanosheets

XPS analysis was introduced to research the valence state of various elements in the nanosheets. The peaks belonging to Mo^4+^ 3d^5/2^ (at 228.6 eV), Mo^6+^ 3d^5/2^ (232.0 eV) and 3d^3/2^ (235.9 eV) (Additional file 1: Fig. S2a); peaks at 74.7 eV and 1304.1 eV were ascribed to Al 2p^3/2^ and Mg 1 s respectively were also detected in the XPS spectrum of Mo element (Additional file 1: Fig. S2b, c). The peak of S^2−^ at 162.18 eV (S 2p^3/2^) was observed as well (Additional file 1: Fig. S2d), confirming that the nanosheets are composed of MoS_2_. In addition, the peaks at 641.2 eV, 653.4 eV, 653.8 eV, and 642.5 eV represent Mn^3+^ 2p ^3/2^, Mn^2+^ 2p ^1/2^, Mn^3+^ 2p^1/2^, and Mn^2+^ 2p ^3/2^ (Fig. 2a), respectively, indicating that the valences of Mn are Mn^3+^ and Mn^2+^. The XPS spectrum of Mn was fitted according to literature and the atom ratio of Mn^3+^ and Mn^2+^ was evaluated to be 50.7:49.3 [44]. The structural nature of LMM nanosheets was then studied using XRD (Fig. 2b). Compared with Mg-Al-LDH, the typical (003), (006), (012) and (110) peaks at 11.70°, 23.54°, 33.4°, 60.23° (JCPDS: 14–0191) were weakened after doping with MoS_2_ (red line: LDH-MoS_2_) and even disappeared after further doping with manganese (blue line: LMM). Besides, the (002) peak of MoS_2_ (JCPDS: 75–1539, red line: LDH-MoS_2_) was also weakened after doping with manganese (blue line: LMM), implying that the doping of Mo and Mn has restricted the growth of the crystalline of Mg-Al-LDH.

FTIR was used to confirm the successful BSA coating (Fig. 2c). The peaks at 3320 cm^−1^ and 2950 cm^−1^ represented the asymmetry elastic of –NH_2_ and –CH_3_ of BSA. Peaks belonging to amide III, II, and I at 1390 cm^−1^, 1540 cm^−1^, and 1640 cm^−1^ could be found in the curve of BSA and LMM@BSA clay nanosheets, revealing the successful coating of BSA. To quantify the amount of surface-coated BSA, LMM, BSA, and LMM@BSA clay nanosheets were programmatically heated to 900 °C in air, which confirms that the mass ratio of surface-coated BSA was approximately 56.4% (Fig. 2d). The size of LMM@BSA clay nanosheets in DMEM was ~ 147 nm, and the size fluctuation of DLS during 24 h was ignorable (Additional file 1: Fig. S3). Moreover, the LMM@BSA clay nanosheets could be well-dispersed in water, PBS, and DMEM and showed the noticeable Tyndall effect (Fig. 1c), indicating that the modified BSA molecules endow the nanosheets with excellent colloidal stability in certain circumstances.

### In vitro photo-thermal performance

**Fig. 3:**
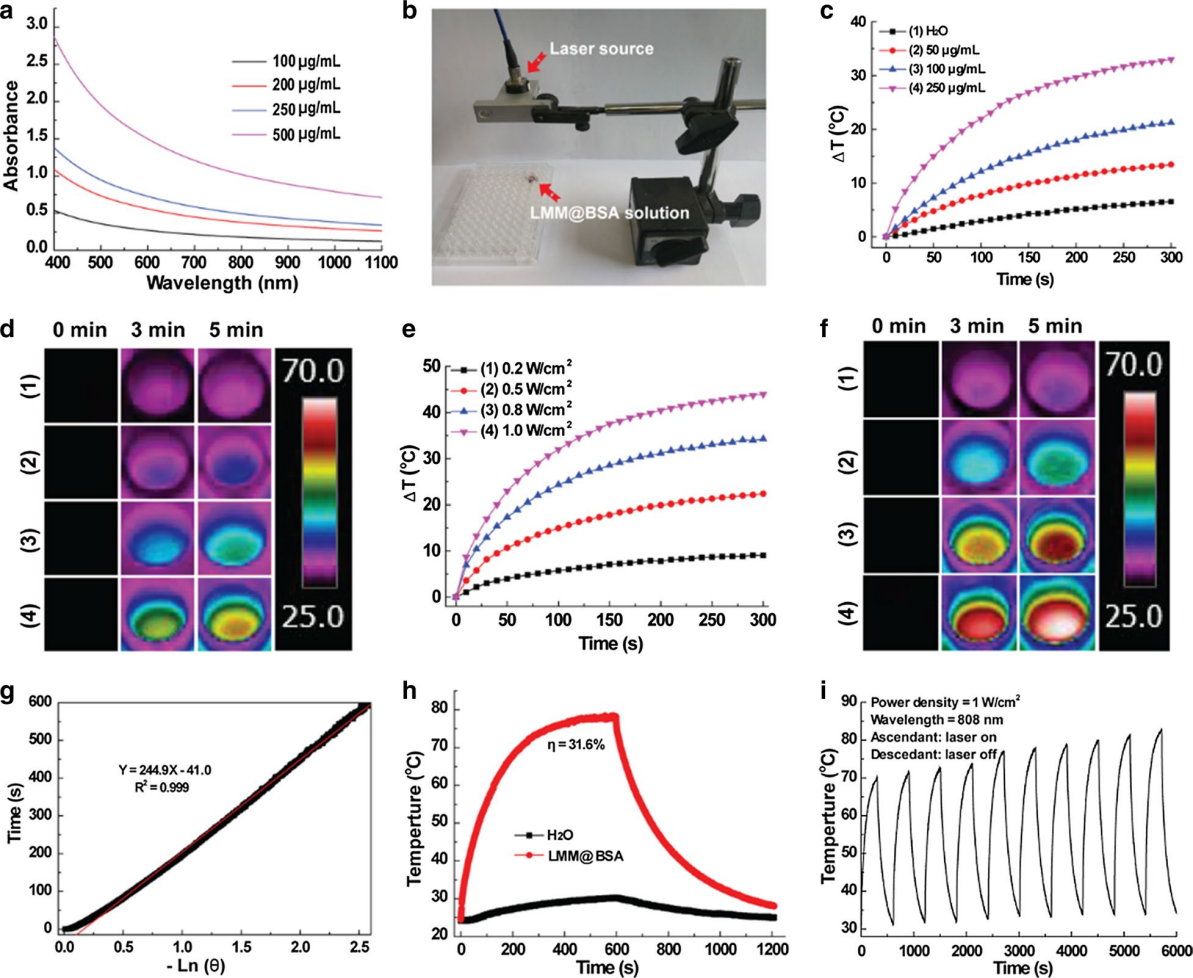
**a** Light absorption of LMM@BSA clay nanosheets solution; **b** the photograph of the experimental appliance; **c** distilled water and concentration-dependent temperature profiles of LMM@BSA clay nanosheets (NIR laser: 1 W/cm^2^); **d** corresponding thermal imaging of **c**; **e** power density-dependent temperature profiles of LMM@BSA clay nanosheets solution; **f** corresponding thermal imaging of **e**; **g** time constant for heat transfer of LMM@BSA clay nanosheets; **h** steady-state heating curve of LMM@BSA clay nanosheets and distilled water and **i** recycling heating profiles of LMM@BSA clay nanosheets. The unit of bar in **d** and **f** is °C

In line with the MoS_2_ [45, 46], the LMM@BSA clay nanosheets demonstrate apparent light absorption that is closely related to their concentration within wavelength from 400 to 1100 nm (Fig. 3a). Setting the experimental device according to Fig. 3b and under laser irradiation (1.0 W/cm^2^, 5 min), the solution temperatures swiftly increased by 13 °C, 21 °C, and 33 °C corresponding to their concentrations at 50 μg/mL, 100 μg/mL, and 250 μg/mL, respectively. However, under the same circumstance, the distilled water merely increased by 6.5 °C (Fig. 3c). When irradiating the LMM@BSA clay nanosheets (500 μg/mL) with varied laser power density, the temperatures increased by 44 °C, 34 °C, 22 °C and 9 °C upon laser powered at 1.0 W/cm^2^, 0.8 W/cm^2^, 0.5 W/cm^2^ and 0.2 W/cm^2^, respectively (Fig. 3e). The related thermal picture captured by the FLIR™ camera further reinforced the relationship of photo-thermal performance with irradiation time, power density and material concentration (Fig. 3d, f). The photo-thermal efficiency of the LMM@BSA clay nanosheets was figured at 31.6% (Fig. 3g, h), which is higher than MoS_2_ nanospheres [16] and other kinds of MoS_2_ based composites (like MoS_2_@Fe_3_O_4_-ICG/Pt(IV) nanoflowers) [15]. Moreover, negligible maximum temperature changes after being irradiated by 808 nm laser for 10 cycles were observed, indicating the desirable thermal stability of LMM@BSA clay nanosheets (Fig. 3i). Given the outstanding photo-thermal conversion efficiency and thermal durability, it was anticipated that LMM@BSA clay nanosheets are suitable for the tumor hyperthermia treatment.

### In vitro compatibility

The appraisal of in vitro cytotoxicity of LMM@BSA clay nanosheets is fundamental for its biomedical applications. The viability of L929 cells that were cultured with LMM@BSA clay nanosheets for 24 h remained higher than 90% (0 – 500 μg/mL, Additional file 1: Fig. S4a), with similar morphology to those treated with saline (control, Additional file 1: Fig. S5a, b). Further, calcein-AM/PI study suggests that the Live/Dead cells staining results were in accordance with CCK-8 assay and the morphology observation evidently proved that the nanosheets would not destruct the integrity of cell membrane in the experimental concentration range (Additional file 1: Fig. S4b–f).

To further prove the hemocompatibility of LMM@BSA, the hemolytic assay was carried out. As calculated, the HPs of experimental samples turned out to be lower than 2% under nanosheets concentration of 0 – 500 μg/mL (Additional file 1: Fig. S6), implying the excellent hemocompatibility of LMM@BSA clay nanosheets within the experimental dosage.

### ***Ce6 loading and detection of ***^***1***^***O***_***2***_

**Fig. 4:**
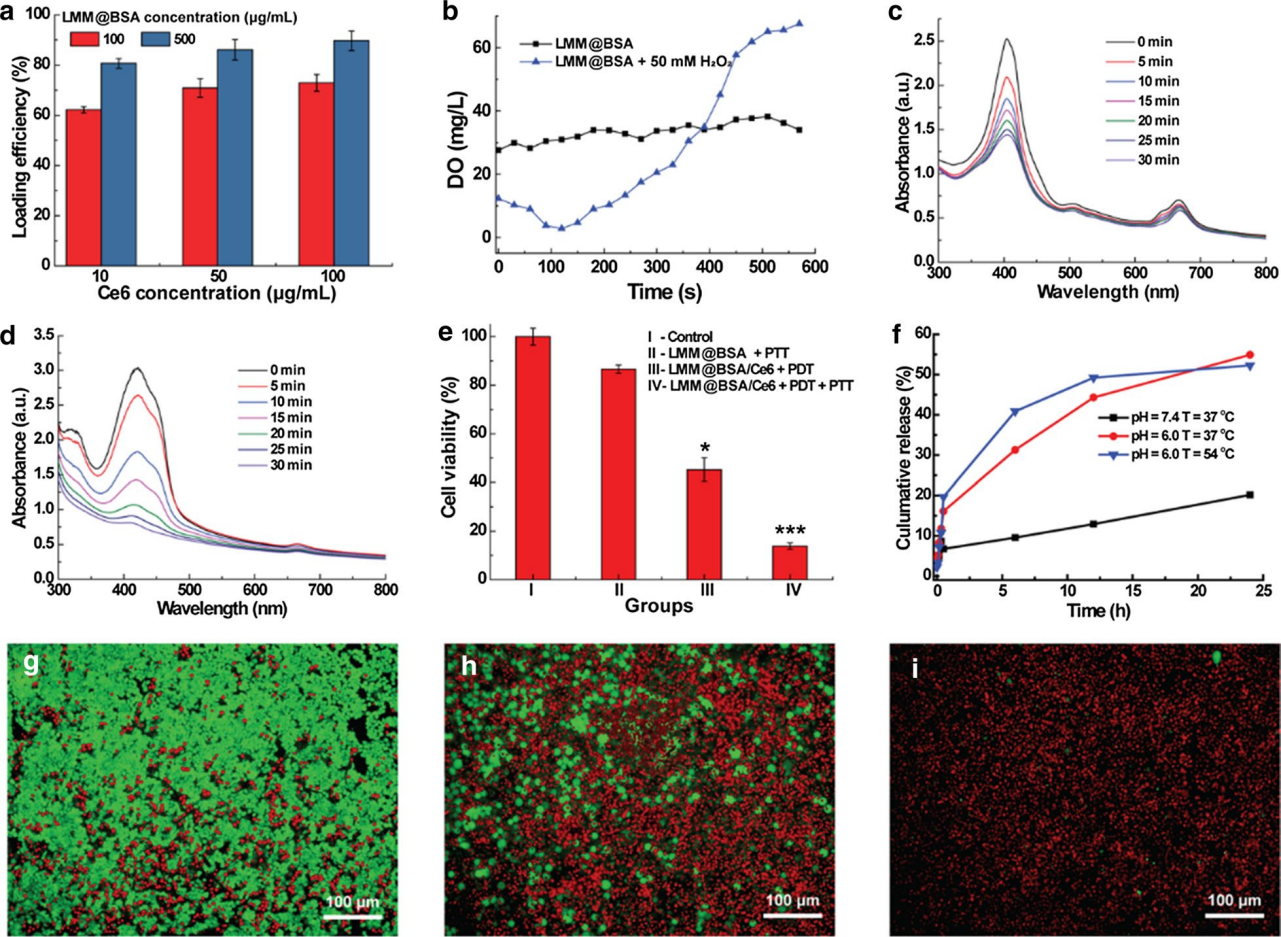
**a** The Ce6 loading efficiency of LMM@BSA clay nanosheets with different concentrations; **b** the DO content (mg/L) of dissolved oxygen in LMM@BSA and LMM@BSA + H_2_O_2_ solution; **c**, **d** absorption spectrum changes of DPBF under 660 nm laser irradiation with LMM@BSA/Ce6 nanosheets **c** in the absence and **d** presence of H_2_O_2_ (10 mM); **e** viability profile of HT29 cells after different treatments; **f** in vitro cumulative release profiles of DOX from LMM@BSA/DOX nanosheets under different pH and temperature; **g**–**i** the calcein-AM/PI dyeing morphology of HT29 cells corresponding to **e**; **g** LMM@BSA + PTT; **h** LMM@BSA/Ce6 + PDT; **i** LMM@BSA/Ce6 + PDT + PTT

The interlayer space and the surface of the LMM@BSA clay nanosheets could contribute to its physical adsorption of drugs or photosensitizers. In this study, the photosensitizer agent, namely Ce6, was mixed with LMM@BSA, after which a characteristic peak at 403 nm belonging to Ce6 was successfully detected in the UV–vis–NIR spectrum of the centrifugal product (Additional file 1: Fig. S7). This indicated that the Ce6 was successfully loaded on LMM@BSA clay nanosheets. Owing to the increased loading sites, the loading efficiency of Ce6 was found to grow with the increasing-concentration of LMM@BSA clay nanosheets. Besides, the increasing of Ce6 concentration could also raise the Ce6 loading efficiency, and a high loading efficiency of 89.37 ± 3.92% was obtained when the LMM@BSA and Ce6 concentrations were 500 μg/mL and 100 μg/mL, respectively (Fig. 4a). The loading percentage of Ce6 was calculated as about 15% when dividing the loaded amount of Ce6 with the total amount of LMM@BSA and Ce6. To confirm the catalytic efficiency of LMM@BSA, the DO content in LMM@BSA solution with and without the addition of H_2_O_2_ was compared. The introduction of H_2_O_2_ led to an increase in the DO content of the LMM@BSA solution (Fig. 4b), implying that the H_2_O_2_ has been transformed into O_2_ under the catalysis of LMM@BSA. It was worth noting that the obvious lower starting value in the DO content might be corresponding to the H_2_O_2_-induced degradation of nanosheets, which consumed the oxygen to some extent.

To certificate that LMM@BSA could enhance the ROS production of Ce6 under the irradiation of 660 nm laser, the ^1^O_2_ production of LMM@BSA/Ce6 was studied (Fig. 4c, d). The structure of DBPF alters quickly upon strong oxidants such as ROS [17], leading to the apparent decrease of light absorption at 410 nm. Therefore, it was applied to indicate the emergence of ^1^O_2_. Upon the irradiation with 660 nm laser, the absorption peaks of DPBF at 410 nm were weakened, implying the continuous generation of ^1^O_2_. Interestingly, the decrease of absorption peak of DPBF at 410 nm was more swiftly after the adding of H_2_O_2_ (Fig. 4d), proving that the ^1^O_2_ production rate was improved. Such a distinct difference in the absorption peaks decreasing-rate of DPBF suggested that the LMM@BSA could trigger the decomposition of H_2_O_2_ to produce O_2_. These dissolved O_2_ molecules took part in the photosensitization of Ce6 and then enhanced the production of ROS.

### In vitro combined tumor therapy

The in vitro tumor PTT efficiency was examined with HT29 cells. Upon the laser irradiation, the viability of HT29 cells decreased with the increasing of LMM@BSA clay nanosheets concentration (0 – 500 μg/mL) and attenuated to 14.2 ± 1.4% when the LMM@BSA clay nanosheets reached 500 μg/mL (Additional file 1: Fig. S8a). By fixing the LMM@BSA at 500 μg/mL, the HT29 cells’ viability declined gradually with the enhancement of power density and a low viability of 14.6 ± 0.1% was found at the laser power density of 1.0 W/cm^2^ (Additional file 1: Fig. S9a). Consistent with the CCK-8 results, the calcein-AM/PI dyeing further demonstrated that the cellular apoptosis relies on nanosheets concentration and laser power density (Additional file 1: Figs. S8b–d and S9b–f), giving more evidence that the NIR laser-caused local hyperthermia could inhibit the cellular proliferation. Based on the concentration and power density-dependent tumor-killing effect, LMM@BSA clay nanosheets with a concentration of 100 μg/mL and 808 nm laser with a power density of 1.0 W/cm^2^ were applied for the synergistic tumor therapy.

With the proved catalytic activity, LMM@BSA clay nanosheets are anticipated to enhance the tumor PDT efficiency after Ce6 loading. To prove this hypothesis, HT29 cells were used as the model to research the in vitro tumor PDT efficiency. The apoptosis extent of HT29 was correlated to the irradiation time, and the cell viability decreased to 40.3 ± 6.0% after 5 min irradiation. The cellular viability of HT29 cells treated with LMM@BSA and LMM@BSA/Ce6 nanosheets decreased to 86.6 ± 1.7% and 41.7 ± 3.3% respectively after the laser irradiation (Fig. 4g–i and Additional file 1: Fig. S10). Interestingly, the cellular viability of HT29 cells attenuated to 13.8 ± 1.3% after successively exposed to 808 nm and 660 nm laser, clearly indicating the combination of tumor PTT and PDT.

### In vivo compatibility

**Fig. 5:**
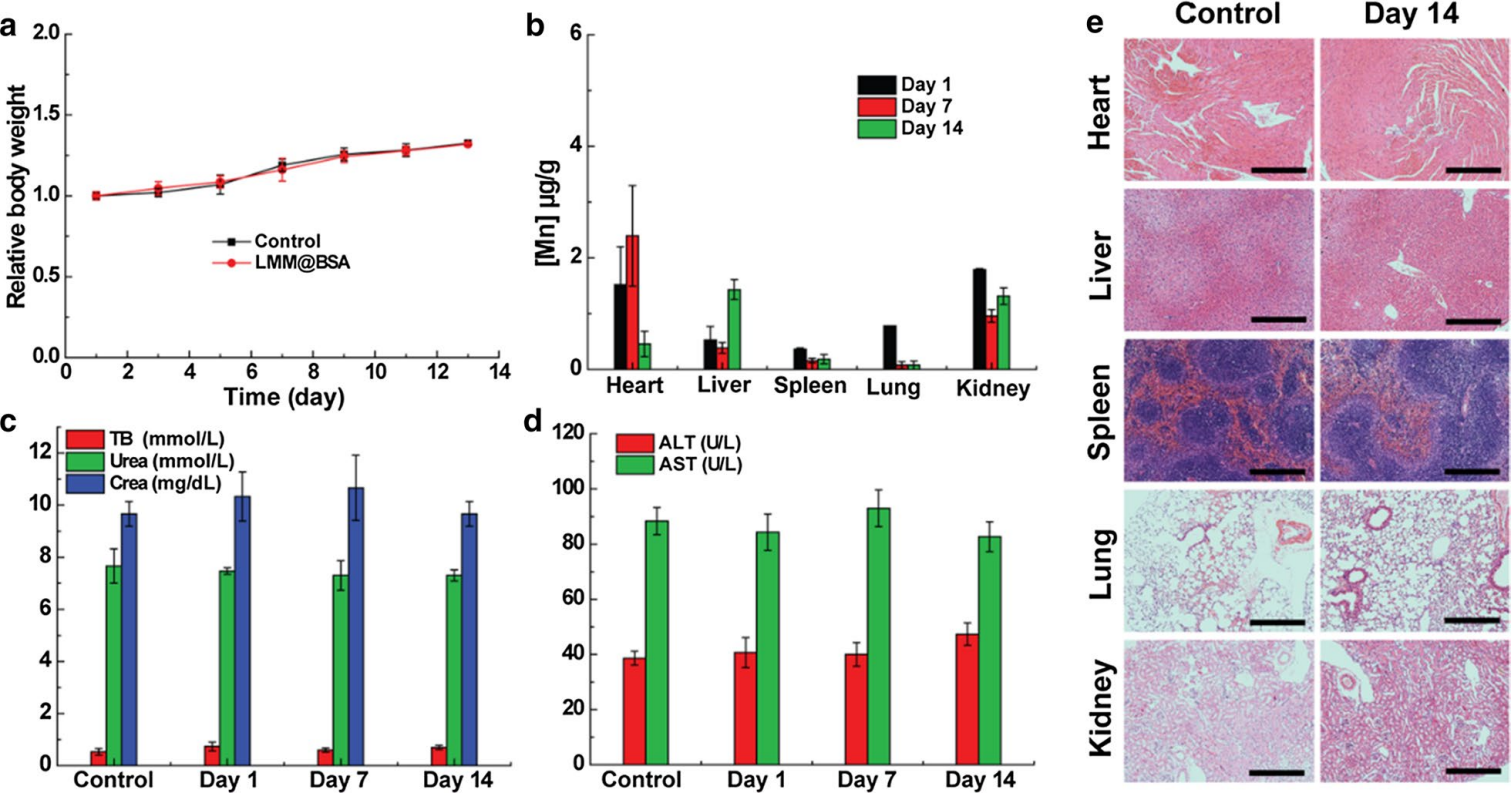
**a** The relative body weight changes of KM mice treated with saline and LMM@BSA clay nanosheets (n = 3); **b** long-term Mn bio-distribution of mice injected with LMM@BSA/Ce6 nanosheets; **c**, **d** serum biochemistry assay of mice injected with saline (control) and LMM@BSA dispersion and fed for different days. *TB* total bilirubin, *CREA* creatinine, *ALT* alanine transaminase, *AST* amino-transferase; **e** H&E-dyed tissue sections of major organs of KM mice that were injected with saline (control) or LMM@BSA clay nanosheets and fed for 14 days (scale bar = 100 μm)

To assess the in vivo biocompatibility, the body weight of mice (I.V. injected with LMM@BSA) was observed, which showed a normal fluctuation during 14 days feeding (Fig. 5a). The routine blood and serum biochemistry tests results displayed negligible statistical differences between the control and the test groups (Fig. 5c, d and Additional file 1: Fig. S11), further revealing the outstanding blood cell safety of the LMM@BSA clay nanosheets. The in vivo biocompatibility was then studied by performing the bio-distribution study of nanosheets. The results proved that the Mn levels in major organs gradually attenuated over the feeding time, indicating that LMM@BSA clay nanosheets could be excreted out of these organs (Fig. 5b). Notably, the Mn content in liver remained a relative high level after 14 days, indicating the in vivo clearance of the injected LMM@BSA was mainly happened in liver. The nanosheets’ long-term biosafety and histocompatibility were finally tracked by H&E dyeing of major organs (Fig. 5e and Additional file 1: Fig. S12). Compared with healthy mice, the LMM@BSA clay nanosheets neither brought any off-target detriment nor pathological effect to normal organs during the feeding, further supporting the excellent biosafety of nanosheets.

### In vitro and in vivo MR imaging

**Fig. 6:**
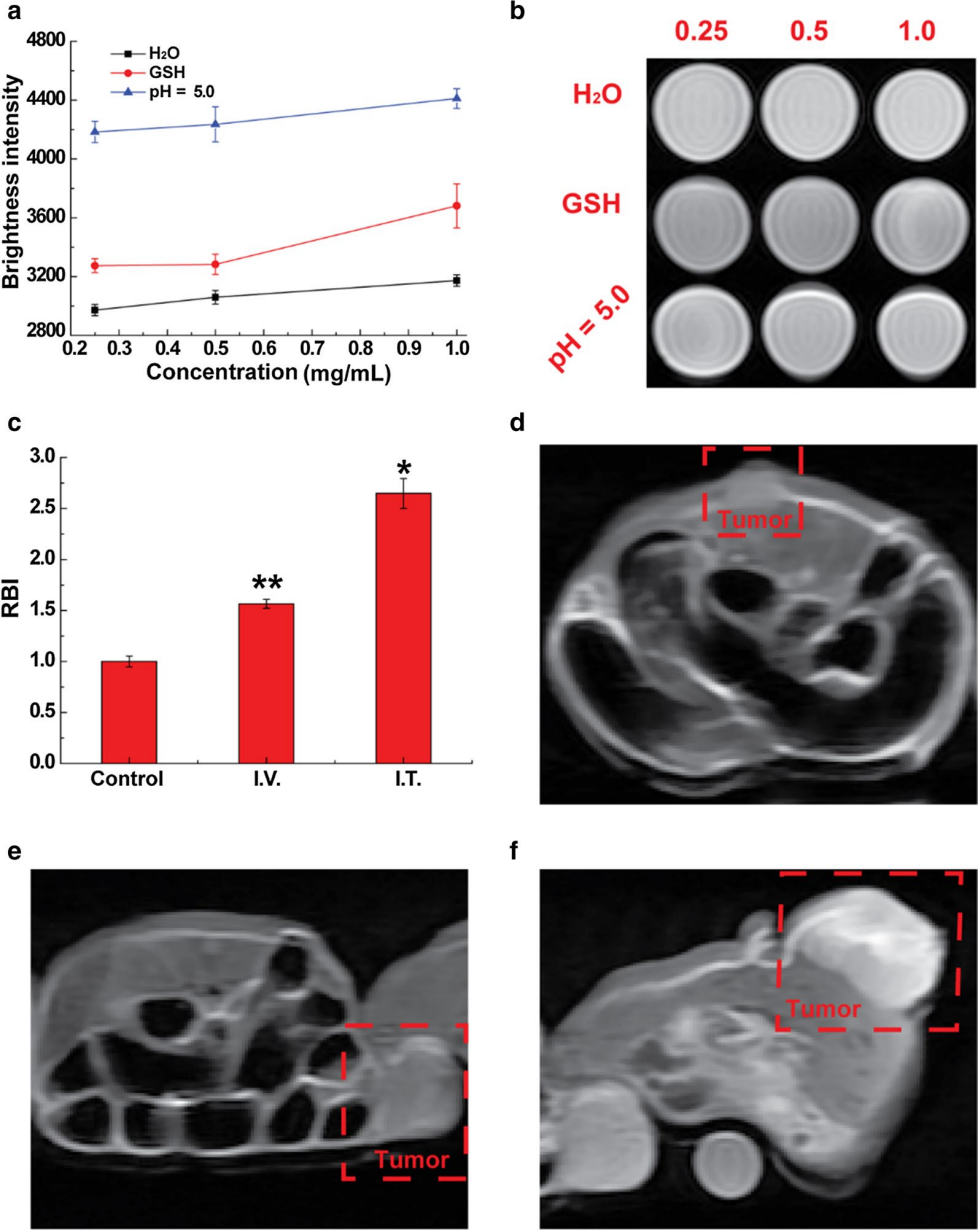
**a** In vitro brightness intensity of LMM@BSA in a mildly acidic environment (pH = 5.0), distilled water or GSH aqueous solutions after soaking at 37 °C for 2 h; **b** T_1_-weighted MR imaging of solution corresponding to **a**, unit: mg/mL; **c** in vivo relative brightness intensity of mice tumor after I.T. or I.V. injected with saline and LMM@BSA; **d**–**f** in vivo T_1_-weighted MR imaging corresponded to **c**, (**d** control, **e** I.V., and **f** I.T.)

Manganese-based nanomaterial has been frequently studied as the imaging contrast for T_1_-weighted MR imaging. Interestingly, such an imaging capacity was well-inherited by the LMM@BSA clay nanosheets. Moreover, the MR imaging is responsive in tumor microenvironment (i.e., mildly acidic and GSH). The brightness intensity of LMM@BSA clay nanosheets increased with its concentration. Surprisingly, owing to its fast break-up in acidic condition, the imaging pictures of nanosheets dispersed in acidic buffer (pH = 5.0) were brighter than in distilled water. The brightness was detected as 4184, 4236 and 4411 when its concentration was 0.25, 0.50, 1.0 mg/mL, respectively in citric acid buffer (pH = 5.0). In distilled water, however, it was only recorded as 2972, 3060 and 3173. Moreover, the solution brightness in GSH increased to 3274, 3283, and 3681, which is higher than that in distilled water, probably owing to the fast-dissolving and reduction of Mn^3+^ to Mn^2+^ by GSH (Fig. 6a, b). The MR imaging capacity of LMM@BSA clay nanosheets was further evaluated in vivo on Balb/c mice that were I.V. or I.T. injected with the LMM@BSA clay nanosheets. A remarkable increase of brightness intensity could be observed within the tumor site after the I.V. (RBI: 1.6, Fig. 6c–e) or I.T. (RBI: 2.7, Fig. 6c, d, f) injection, which provides the powerful proof for the promising in vivo MR imaging and diagnosing of tumor.

### In vivo combined tumor therapy

**Fig. 7:**
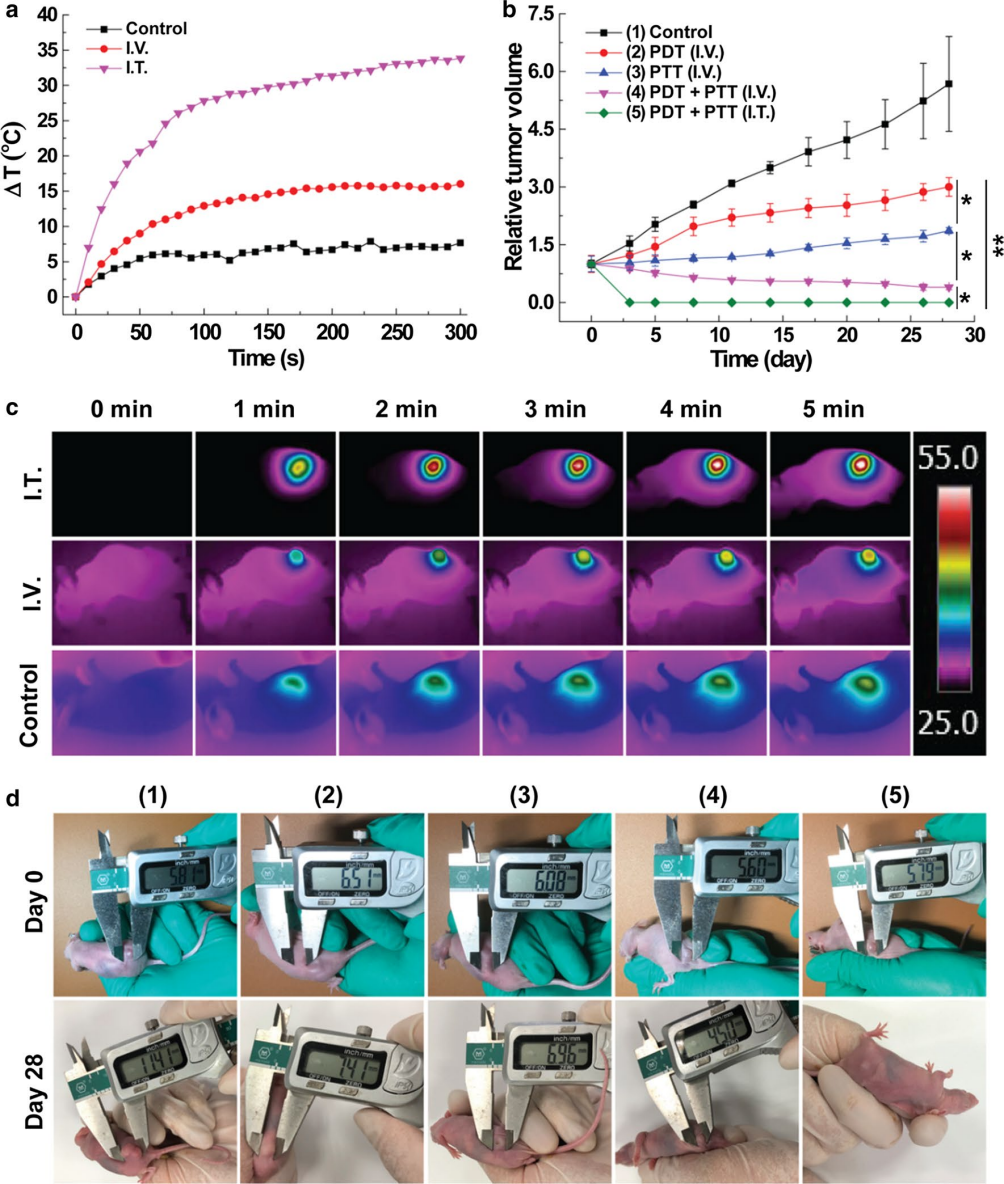
**a** The tumor heating curves of mice under NIR laser irradiation; **b** tumor growth profile of mice after various treatments as noted; **c** in vivo thermal imaging of mice after laser irradiation for different time points corresponding to **a**; **d** pictures of HT29 tumor-bearing mice at day 0 and day 28 corresponding to **b**. The unit of the bar in **c**: °C

The tumor treatment effectiveness of LMM@BSA/Ce6 nanosheets was finally verified on tumor-bearing mice (HT29 colorectal carcinoma, Fig. 7). Due to the photo-thermal efficiency, the tumor surface temperature of the mice with I.V. or I.T. LMM@BSA injection raised by ~ 16 °C or ~ 33 °C upon the 5 min 808 nm laser irradiation. However, no noticeable temperature changes were found in the control group (Fig. 7a). In vivo imaging of mice further proved the photo-thermal effect of LMM@BSA and the neglectable thermal effect of saline (Fig. 7c). Subsequently, the therapy efficiency was monitored by measuring the tumor volume and recording digital photographs (Fig. 7b, d). The tumor volume of the control group was about 5.7 ± 1.2 times larger than the original after 28 days feeding. After single laser irradiation, the tumor expanded 3.0 ± 0.2 (660 nm) and 1.9 ± 0.1 times (808 nm) of its volume after 28 days feeding, manifesting that the individual PTT or PDT therapy could inhibit the tumor growth to some extent. Notably, the tumor volume shrank to approximate 40% of the original (I.V. injection) and were eradicated (I.T. injection) after the combined PDT and PTT, visually illustrating that the combination of hyperthermia and ROS produced the excellent tumor restraining-effect.

### Drug loading and releasing behavior

In addition to Ce6, the LMM@BSA clay nanosheets could also work as a carrier to physically load antitumor drugs (e.g., DOX) and control its release. The optimized DOX loading efficiency was calculated as 66.9%. The drug release pattern was plotted by changing external conditions. The releasing rate was lower than 10% (6.2%) in neutral condition (50 °C). However, it was significantly improved in the acidic condition (pH = 6.0), which reached 54.9% and 52.3% under 37 °C and 54 °C, respectively (Fig. 4e). The DOX was also released more swiftly in higher temperature, meaning that the NIR would promote the drug-releasing. This DOX releasing kinetics with pH and temperature (NIR laser)-dependent properties could equip the LMM@BSA clay nanosheets with competitiveness for tumor chemotherapy, which would be discussed in the future study.

## Conclusion

In summary, a novel kind of biocompatible Mg–Mn–Al LDH-based nano-platform, namely LMM@BSA which could integrate the photo-thermal effect of MoS_2_ was synthesized using a hydrothermal approach. The surface of LMM was coated with BSA to render it with excellent colloidal stability under physiological conditions. Owing to the fast break-up in acidic conditions and the reduction of Mn^3+^ to Mn^2+^ by GSH in tumor micro-environment, the MR imaging of Mg–Mn–Al LDH was strengthened. Notably, the interlayer space and the surface of LMM@BSA clay nanosheets could be used to efficiently load Ce6. The ROS production of Ce6 under the light irradiation was distinctively enhanced since the LMM@BSA could catalyze the decomposing of H_2_O_2_ in the tumor to produce oxygen. In addition, the high photo-thermal conversion efficiency of the MoS_2_ component could elevate the local temperature of tumor, which successfully achieved a remarkable PTT and PDT combined anticancer outcome. Compared with other kinds of nanocarriers, the LMM holds a more promising future to carry guest molecules since it has not only the interlayer space, but also the unique 2D morphology. Although this synthetic LMM@BSA is not degradable, this work presents an advances in the rational design of LDH-based cancer-therapeutic modalities with simultaneous low side effects and high therapeutic results.

## Supplementary Information

**Additional file 1: Figure S1.** (a) AFM image of LMM@BSA, (b) the height of LMM@BSA. **Figure S2.** XPS spectra of LMM: (a) Mo 3d, (b) Al 2p, (c) Mg 1s and (d) S 2p. **Figure S3.** Dynamic light scattering of LMM@BSA nanosheets dispersed in DMEM. **Figure S4.** (a) Cell viability profiles; (b–f) appearance of calcein-AM/PI dyed L929 cells treated with different concentration of LMM@BSA clay nanosheets (incubation time: 24 h): (b) control; (c) 50 μg/mL; (d) 100 μg/mL; (e) 250 μg/mL; (f) 500 μg/mL. **Figure S5.** (a, b) The morphology of cell treated with (a) saline and (b) LMM@BSA clay nanosheets (500 μg/mL). **Figure S6. **Hemolysis percentage of mRBCs co-incubated with LMM@BSA clay nanosheets (concentration: 0–500 μg/mL) for 2 h. **Figure S7. **UV–vis–NIR spectra of prepared nanosheets. **Figure S8.** (a) Cell viability profiles; (b–d) appearance of calcein-AM/PI dyed cells after PTT treatments (1 W/cm^2^) with different concentration of LMM@BSA clay nanosheets: (b) 100 μg/mL; (c) 250 μg/mL; (d) 500 μg/mL. **Figure S9.** (a) Cell viability profiles after different treatments; (b–g) appearance of calcein-AM/PI stained cells treated with (b) DMEM + saline without laser; (c) DMEM + LMM@BSA without laser; (d–g) DMEM + LMM@BSA with laser of (d) 0.2 W/cm^2^; (e) 0.5 W/cm^2^; (f) 0.8 W/cm^2^; (g) 1.0 W/cm^2^. Control: 0 2 W/cm^2^. **Figure S10.** (a) Cell viability profiles treated with LMM@BSA/Ce6 annd 660 nm laser (0.1 W/cm^2^) irradiation for different time points; (b–f) appearance of calcein-AM/PI stained cells after PDT treatments with varied irradiated duration: (b) 0 min; (c) 1 min; (d) 2 min; (e) 3 min; (f) 5 min. **Figure S11.** The routine blood test of mice (a) white blood cell (WBC); (b) red blood cells (RBC); (c) hemoglobin (HGB); (d) hematocrit (HCT); (e) mean corpuscular volume (MCV); (f) mean corpuscular hemoglobin (MCH); (g) mean corpuscular hemoglobin concentration (MCHC); (h) platelet (PLT); and (i) red cell distribution width (RDW) with feeding for varied days. **Figure S12. **H&E-stained tissue sections of major organs of KM mice that injected with saline or LMM@BSA/Ce6 nanosheets and fed for 1 and 7 days.
