# Supplementary material for: 2D LDH-MoS2 clay nanosheets: synthesis, catalase-mimic capacity, and imaging-guided tumor photo-therapy
Source: J Nanobiotechnology. 2021 Feb 3;19:36. doi: 10.1186/s12951-020-00763-7 (PMC7860036; doi:10.1186/s12951-020-00763-7)
Supplement: Supplementary file 1 — Additional file 1: Figure S1. (a) AFM image of LMM@BSA, (b) the height of LMM@BSA. Figure S2. XPS spectra of LMM: (a) Mo 3d, (b) Al 2p, (c) Mg 1s and (d) S 2p. Figure S3. Dynamic light scattering of LMM@BSA nanosheets dispersed in DMEM. Figure S4. (a) Cell viability profiles; (b–f) appearance of calcein-AM/PI dyed L929 cells treated with different concentration of LMM@BSA clay nanosheets (incubation time: 24 h): (b) control; (c) 50 μg/mL; (d) 100 μg/mL; (e) 250 μg/mL; (f) 500 μg/mL. Figure S5. (a, b) The morphology of cell treated with (a) saline and (b) LMM@BSA clay nanosheets (500 μg/mL). Figure S6. Hemolysis percentage of mRBCs co-incubated with LMM@BSA clay nanosheets (concentration: 0–500 μg/mL) for 2 h. Figure S7. UV–vis–NIR spectra of prepared nanosheets. Figure S8. (a) Cell viability profiles; (b–d) appearance of calcein-AM/PI dyed cells after PTT treatments (1 W/cm2) with different concentration of LMM@BSA clay nanosheets: (b) 100 μg/mL; (c) 250 μg/mL; (d) 500 μg/mL. Figure S9. (a) Cell viability profiles after different treatments; (b–g) appearance of calcein-AM/PI stained cells treated with (b) DMEM + saline without laser; (c) DMEM + LMM@BSA without laser; (d–g) DMEM + LMM@BSA with laser of (d) 0.2 W/cm2; (e) 0.5 W/cm2; (f) 0.8 W/cm2; (g) 1.0 W/cm2. Control: 0 2 W/cm2. Figure S10. (a) Cell viability profiles treated with LMM@BSA/Ce6 annd 660 nm laser (0.1 W/cm2) irradiation for different time points; (b–f) appearance of calcein-AM/PI stained cells after PDT treatments with varied irradiated duration: (b) 0 min; (c) 1 min; (d) 2 min; (e) 3 min; (f) 5 min. Figure S11. The routine blood test of mice (a) white blood cell (WBC); (b) red blood cells (RBC); (c) hemoglobin (HGB); (d) hematocrit (HCT); (e) mean corpuscular volume (MCV); (f) mean corpuscular hemoglobin (MCH); (g) mean corpuscular hemoglobin concentration (MCHC); (h) platelet (PLT); and (i) red cell distribution width (RDW) with feeding for varied days. Figure S12. H&E-stained tissue sections of maj [file 12951_2020_763_MOESM1_ESM.docx]

**Additional file 1**

**2D LDH-MoS_2_ clay nanosheets: synthesis, catalase-mimic capacity, and imaging-guided tumor photo-therapy**

Jiayan Zhao,^a,b,1^ Hang Wu,^c,1^ Jiulong Zhao,^a^ Yichen Yin,^b^ Zhilun Zhang,^b^ Shige Wang,^b^ and Kun Lin^a^*

^a^ Department of Gastroenterology, Changhai Hospital, Second Military Medical University, No. 168 Changhai Road, Shanghai, 200433, P. R. China

^b^ College of Science, University of Shanghai for Science and Technology, No. 334 Jungong Road, Shanghai, 200093, P. R. China

^c^ Department of General surgery ,Xinhua Hospital, Shanghai jiaotong University school of medicine , No. 1665 Kongjiang Road, Shanghai, 200433, P. R. China

^1^ These authors contributed equally to this work. *To whom correspondence should be addressed email: lin17321171461@163.com (Mr. Lin)


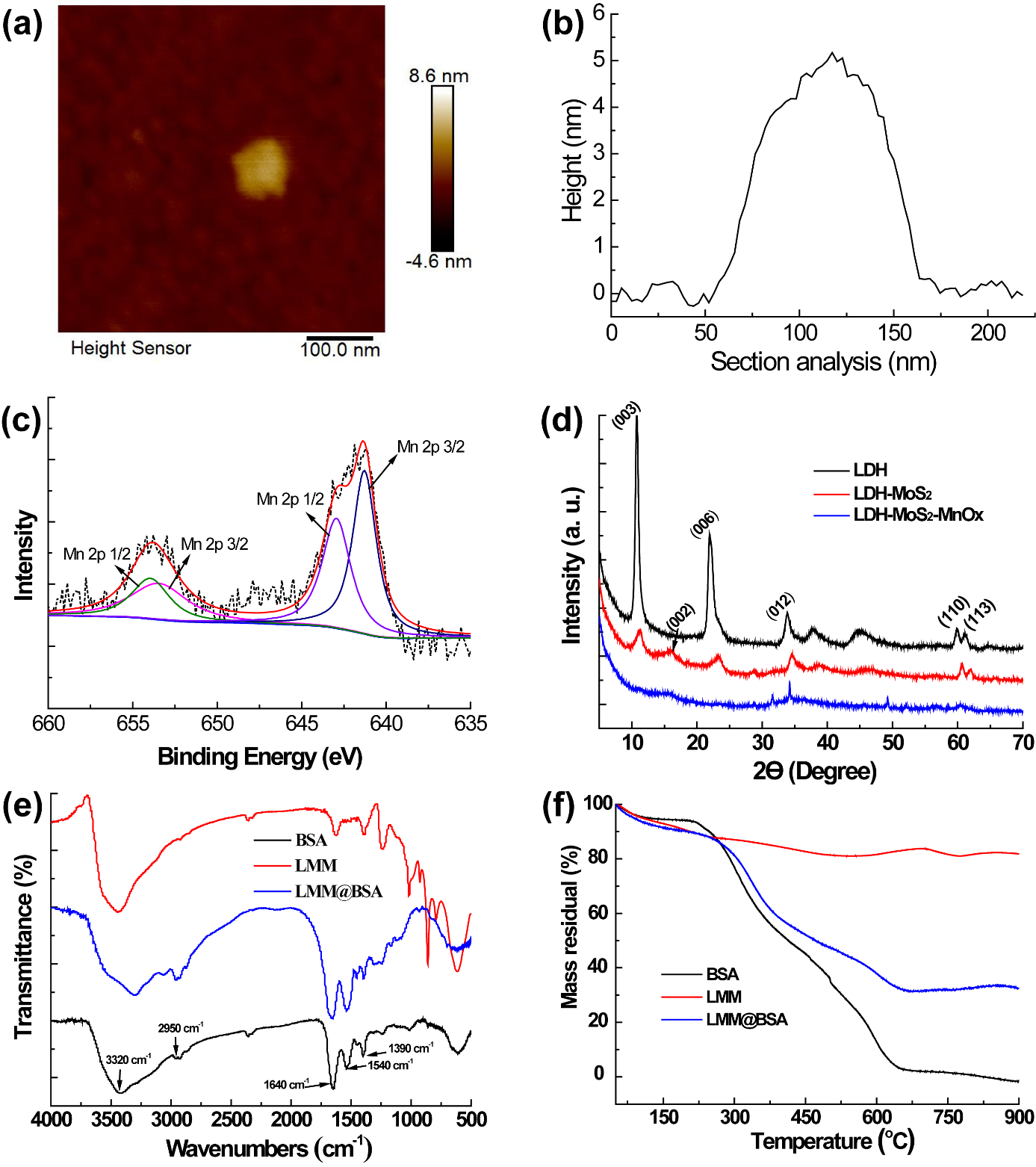


**Figure S1** (a) AFM image of LMM@BSA, (b) the height of LMM@BSA.


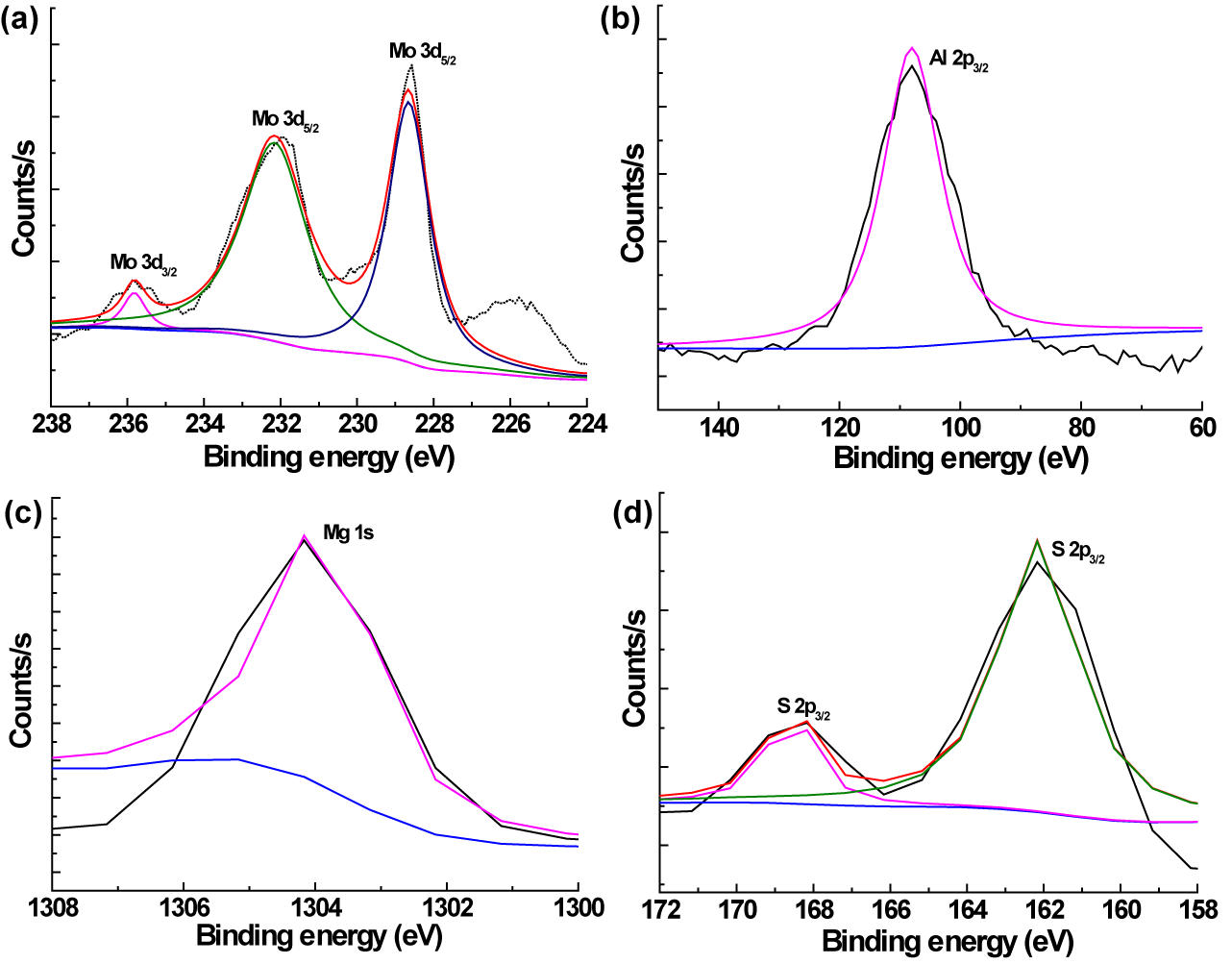


**Figure S2** XPS spectra of LMM: (a) Mo 3d, (b) Al 2p, (c) Mg 1s and (d) S 2p.


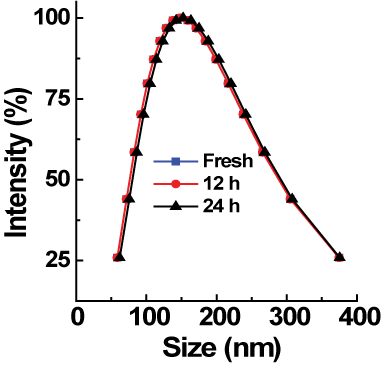


**Figure S3** Dynamic light scattering of LMM@BSA nanosheets dispersed in DMEM.


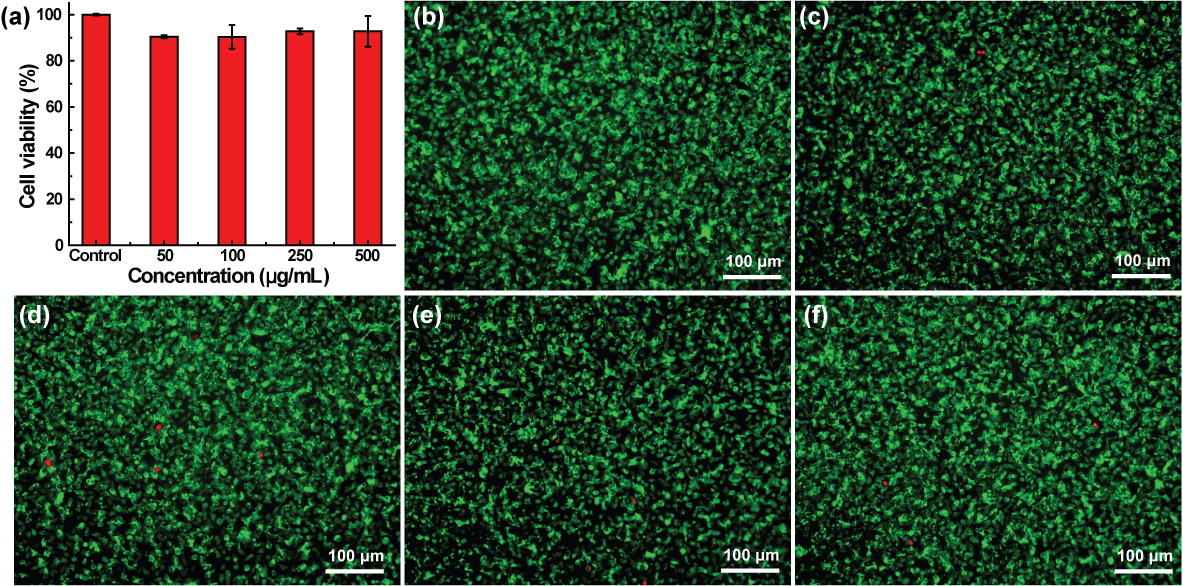


**Figure S4** (a) Cell viability profiles; (b-f) appearance of calcein-AM/PI dyed L929 cells treated with different concentration of LMM@BSA clay nanosheets (incubation time: 24h): (b) control; (c) 50 μg/mL; (d) 100 μg/mL; (e) 250 μg/mL; (f) 500 μg/mL.


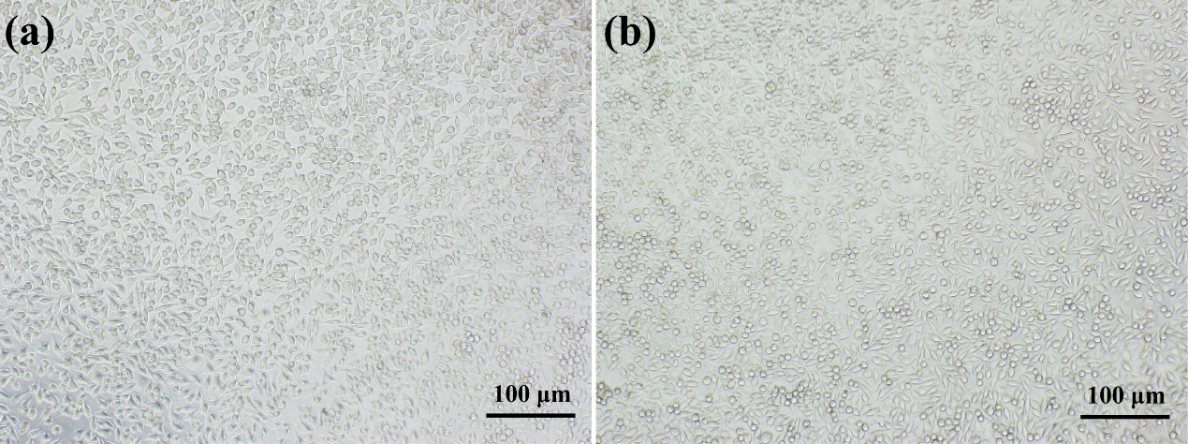


**Figure S5** (a-b) The morphology of cell treated with (a) saline and (b) LMM@BSA clay nanosheets (500 μg/mL).


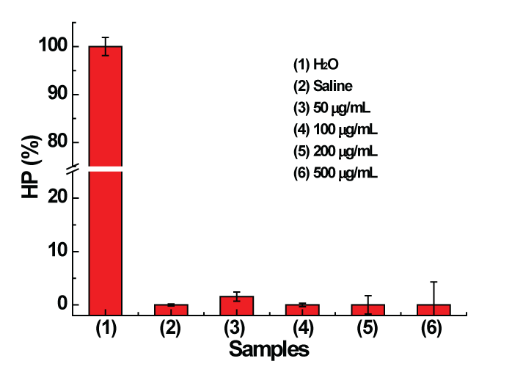


**Figure S6** Hemolysis percentage of mRBCs co-incubated with LMM@BSA clay nanosheets (concentration: 0-500 μg/mL) for 2 h.


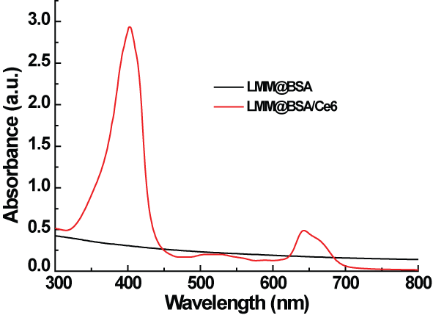


**Figure S7** UV-vis-NIR spectra of prepared nanosheets.


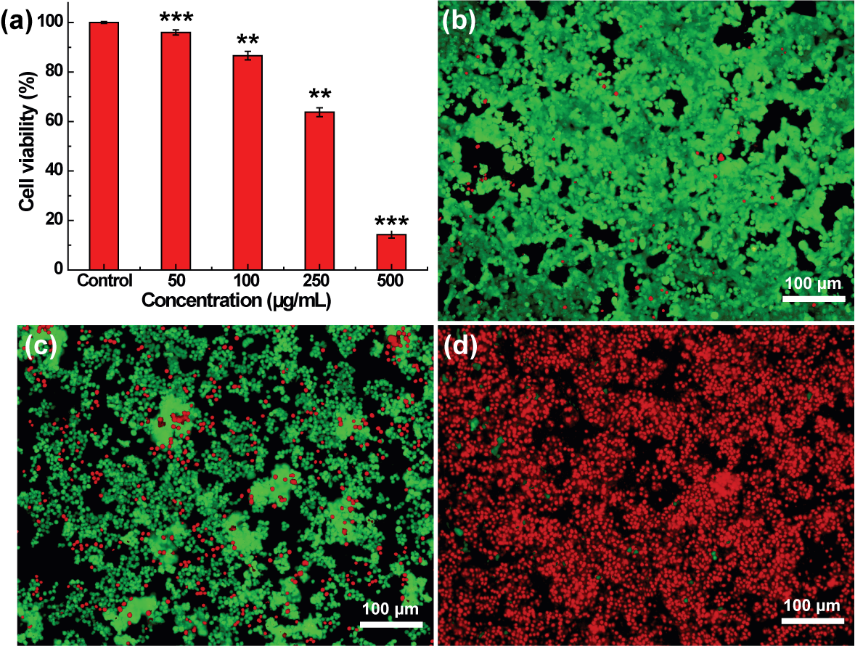


**Figure S8** (a) Cell viability profiles; (b-d) appearance of calcein-AM/PI dyed cells after PTT treatments (1 W/cm^2^) with different concentration of LMM@BSA clay nanosheets: (b) 100 μg/mL; (c) 250 μg/mL; (d) 500 μg/mL.


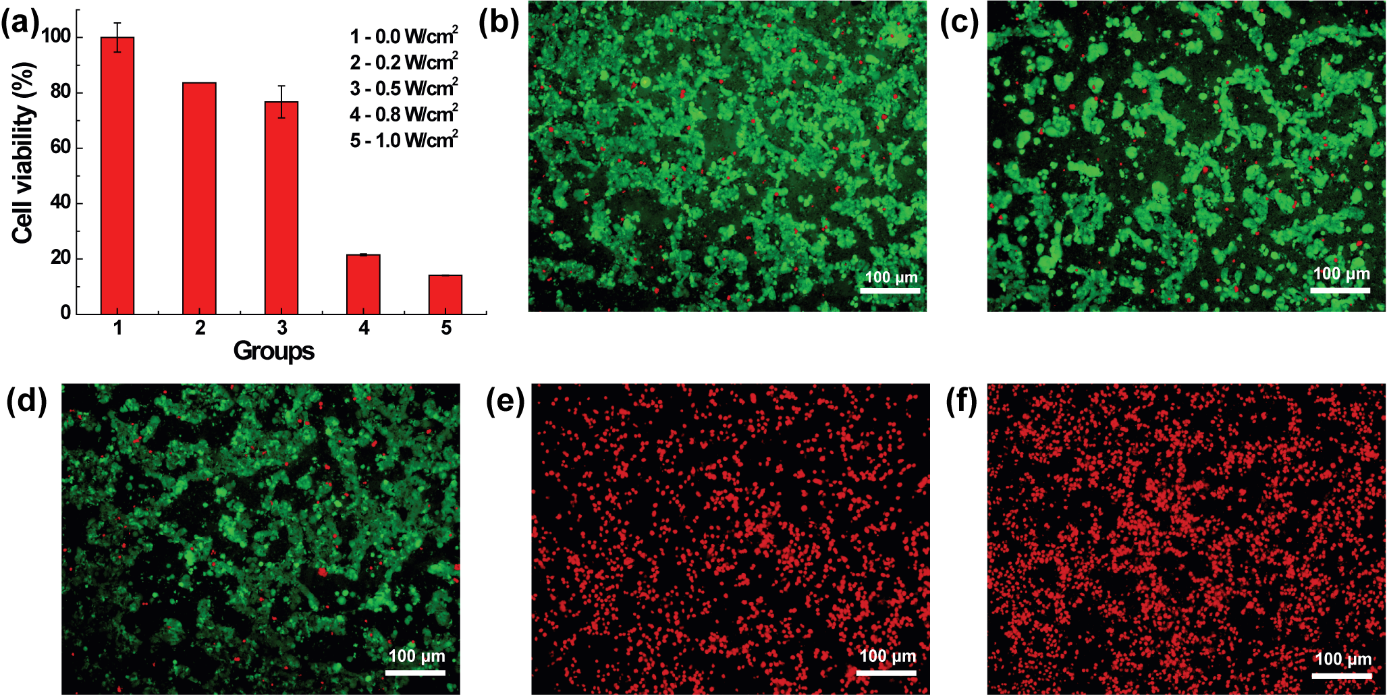


**Figure S9** (a) Cell viability profiles after different treatments; (b-g) appearance of calcein-AM/PI stained cells treated with (b) DMEM + saline without laser; (c) DMEM + LMM@BSA without laser; (d-g) DMEM + LMM@BSA with laser of (d) 0.2 W/cm^2^; (e) 0.5 W/cm^2^; (f) 0.8 W/cm^2^; (g) 1.0 W/cm^2^. Control: 0 2 W/cm^2^.


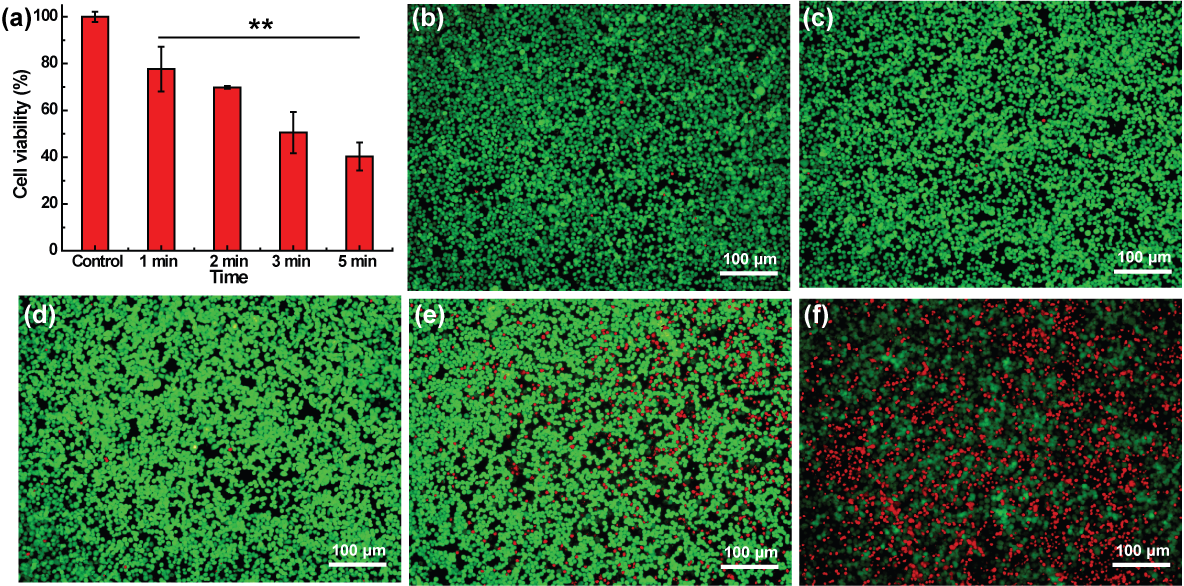


**Figure S10** (a) Cell viability profiles treated with LMM@BSA/Ce6 annd 660 nm laser (0.1 W/cm^2^) irradiation for different time points; (b-f) appearance of calcein-AM/PI stained cells after PDT treatments with varied irradiated duration: (b) 0 min; (c) 1 min; (d) 2 min; (e) 3 min; (f) 5 min.


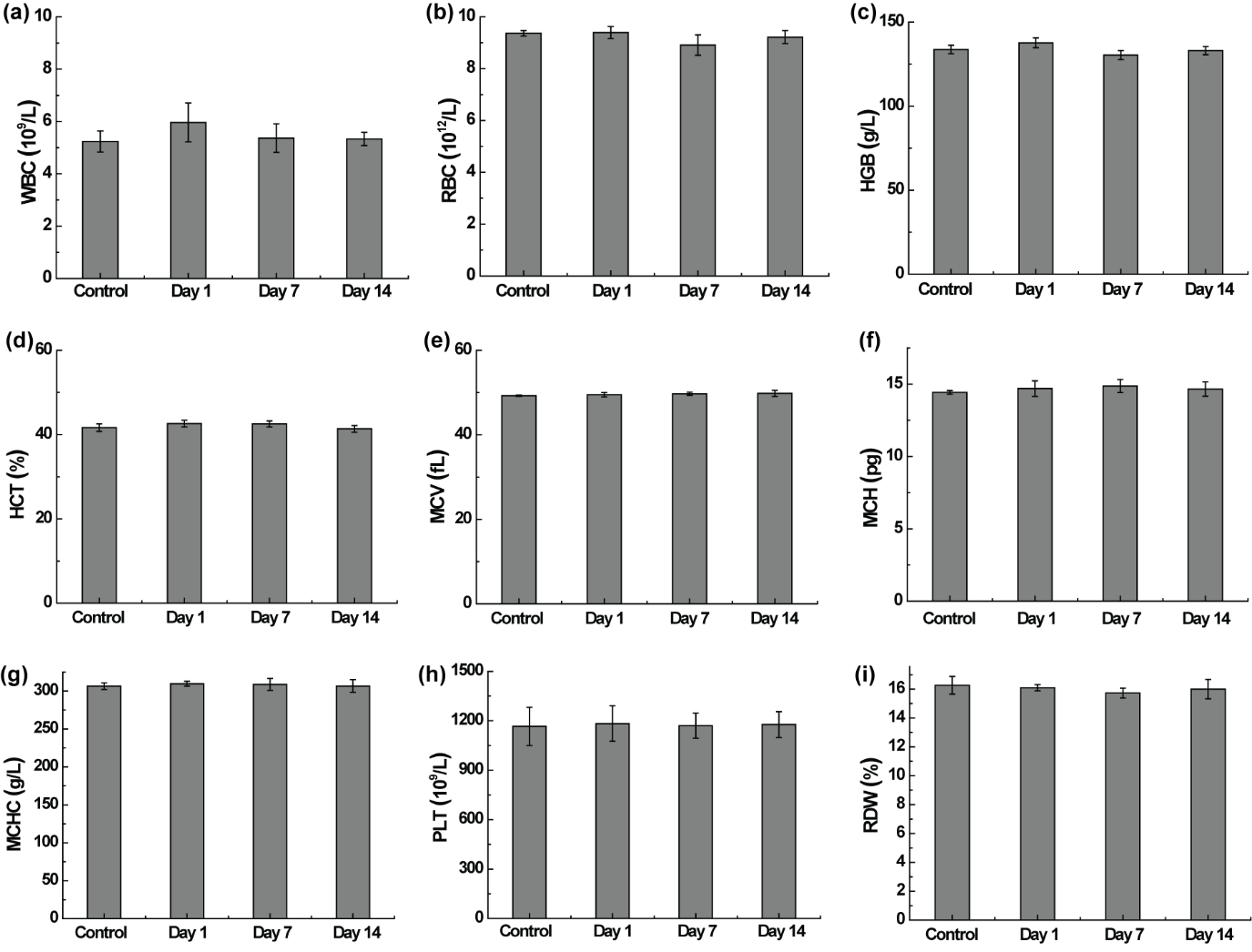


**Figure S11** The routine blood test of mice (a) white blood cell (WBC); (b) red blood cells (RBC); (c) hemoglobin (HGB); (d) hematocrit (HCT); (e) mean corpuscular volume (MCV); (f) mean corpuscular hemoglobin (MCH); (g) mean corpuscular hemoglobin concentration (MCHC); (h) platelet (PLT); and (i) red cell distribution width (RDW) with feeding for varied days.


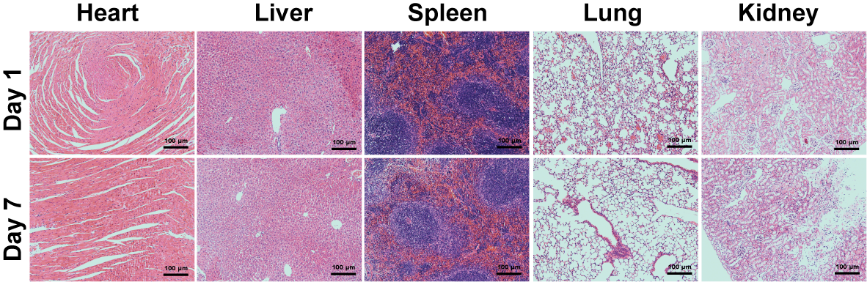


**Figure S12** H&E-stained tissue sections of major organs of KM mice that injected with saline or LMM@BSA/Ce6 nanosheets and fed for 1 and 7 days.
